# Laricitrin 3-Rutinoside from *Ginkgo biloba* Fruits Prevents Damage in TNF-α-Stimulated Normal Human Dermal Fibroblasts

**DOI:** 10.3390/antiox12071432

**Published:** 2023-07-15

**Authors:** Sullim Lee, Yea Jung Choi, Chen Huo, Akida Alishir, Ki Sung Kang, Il-Ho Park, Taesu Jang, Ki Hyun Kim

**Affiliations:** 1 Department of Life Science, College of Bio-Nano Technology, Gachon University, Seongnam 13120, Republic of Korea; sullimlee@gachon.ac.kr; 2 College of Korean Medicine, Gachon University, Seongnam 13120, Republic of Korea; domdada22@gachon.ac.kr (Y.J.C.); kkang@gachon.ac.kr (K.S.K.); 3 School of Pharmacy, Sungkyunkwan University, Suwon 16419, Republic of Korea; huochen_0213@163.com (C.H.); akida.alishir@gmail.com (A.A.); 4 College of Pharmacy, Sahmyook University, 815, Hwarang-ro, Nowon-gu, Seoul 01795, Republic of Korea; parkilho@syu.ac.kr; 5 Health Administration, Dankook University, Cheonan 31116, Republic of Korea

**Keywords:** *Ginkgo biloba* fruits, laricitrin 3-rutinoside, normal human dermal fibroblasts, TNF-α, skin aging

## Abstract

Human skin comprises the epidermis and dermis, which perform interactive functional activities with each other in order to maintain the skin’s tensile strength. In particular, the dermal layer is crucial for skin protection. However, skin aging destroys collagen and elastin fibers, causing wrinkles, pigments, and sagging. Skin aging-related factors, such as tumor necrosis factor-α (TNF-α), promote the generation of intercellular reactive oxygen species (ROS). These are known to stimulate the hypersecretion of matrix metalloproteinase-1 (MMP-1), which degrades collagen and inhibits collagen synthesis. In this study, as part of our ongoing discovery of natural products, we investigated potential natural products derived from ginkgo fruit (*Ginkgo biloba* fruit) with protective effects against TNF-α-induced skin aging. Phytochemical investigation of the MeOH extract of *G. biloba* fruits, aided by liquid chromatography–mass spectrometry, led to the isolation of 14 compounds (**1**–**14**) from the *n*-butanol-soluble fraction. These were structurally determined to be: (*E*)-coniferin (**1**), syringin (**2**), 4-hydroxybenzoic acid 4-O-β-D-glucopyranoside (**3**), vanillic acid 4-O-β-D-glucopyranoside (**4**), glucosyringic acid (**5**), (*E*)-ferulic acid 4-O-β-D-glucoside (**6**), (*E*)-sinapic acid 4-O-β-D-glucopyranoside (**7**), ginkgotoxin-5-glucoside (**8**), ginkgopanoside (**9**), (*Z*)-4-coumaric acid 4-O-β-D-glucopyranoside (**10**), (1′*R*,2′*S*,5′*R*,8′*S*,2′*Z*,4′*E*)-dihydrophaseic acid 3’-O-β-D-glucopyranoside (**11**), eucomic acid (**12**), rutin (**13**), and laricitrin 3-rutinoside (L3R) (**14**). Biological evaluation of the isolated compounds for their effects on intracellular ROS generation showed that, of these 14 compounds, L3R (**14**) inhibited TNF-α-stimulated ROS generation (*p* < 0.001 at 100 μM). Inhibition of ROS generation by L3R led to the suppression of MMP-1 secretion and protection against collagen degradation. The inhibitory effect of L3R was mediated by the inhibition of extracellular signal regulated kinase (ERK) phosphorylation. Furthermore, L3R diminished the secretion of pro-inflammatory cytokines interleukin 6 (IL-6) and interleukin 8 (IL-8). Based on these experimental results, L3R is a potential bioactive natural product that can be used to protect against skin damage, including aging, in cosmetics and pharmaceuticals.

## 1. Introduction

The human skin is a primary barrier network matrix spanning the entire body, functioning to protect interior organs [1]. Skin is comprised of the epidermis and dermis layers, which interact with each other. As the outermost layer of the skin, the epidermis contains avascular tissue, whereas the dermis contains vascular tissue that provides nutrition to the epidermis [2]. The dermis is constructed from an extracellular matrix (ECM) composed of collagen and elastin. These components maintain skin’s strength and its tensile properties [3]. Collagen and elastin are secreted by dermal fibroblasts [4] and their components are destroyed by the aging process. Skin aging is caused by various exogenous and endogenous factors. Endogenous aging occurs inside the human body due to personal gene characteristics and changes in hormone levels [5,6]. Exogenous aging is triggered by external environmental factors such as ultraviolet (UV) radiation. UV radiation is the primary cause of exogenous aging; it induces diverse skin aging factors, including reactive oxygen species (ROS) and the pro-inflammatory factor tumor necrosis factor (TNF)-α [7,8]. TNF-α causes an inflammatory reaction that promotes oxidative stress in the mitochondria; this, in turn, induces intracellular generation of ROS [9], which triggers collagenase synthesis. Collagenases, such as MMP-1 and MMP-9, are known to degrade collagen and inhibit its synthesis [10,11]. Meanwhile, ROS induce various molecular pathways related to skin aging, and increase MAPKs phosphorylation; this upregulates the transcription factors activator protein 1 (AP-1) and nuclear factor-kappa B (NF-κB) [12]. These pathways affect gene expression, increasing the expression of collagenase and pro-inflammatory cytokines [13,14]. Accordingly, we investigated natural products with potential protective effects against TNF-α-induced skin aging.

*Ginkgo biloba* L. (Ginkgoaceae), one of the most ancient medicinal plants, is native to China, and is also distributed throughout Korea and Japan [15,16,17]. Evolutionarily, *G. biloba* has long been termed a “living fossil”, as it is the single surviving species of the Ginkgo family [18]. *G. biloba* has been widely used as a traditional medicine for more than 200 million years, and is thought to provide a variety of therapeutic benefits to living organisms, such as the treatment of asthma, nervousness, stomach discomfort, ischemic heart disease, hearing loss, anxiety, and diabetes mellitus [18,19]. In addition, *G. biloba* is a well-known botanical dietary supplement worldwide and contains several types of bioactive components, including flavonoids, terpenes, polysaccharides, fatty acids, proanthocyanidins, and trilactones [20,21,22]. Modern pharmacological studies have revealed that *G. biloba* exhibits biological activities, including antioxidant, anti-depressant, neurodegenerative diseases, and hepatoprotective effects [23,24]. 

The prolific chemical and pharmacological studies of the extract of *G. biloba* leaves have received extensive attention, and it has been widely proven that the leaf extract can treat cardiovascular and neurological disorders [25,26,27,28]. Considering these useful pharmacological properties, our group has aimed to explore potential bioactive compounds from the extracts of *G. biloba* leaves. As a result, we recently reported two undescribed phenolic compounds (ginkwanghols A and B) and one diarylpentanoid (ginkgobilol) and found that all three compounds promoted osteogenic differentiation by first promoting the mRNA expression of the osteogenic markers ALP and osteopontin [29,30]. 

Moreover, it has been highlighted that the fruits of the *G. biloba* tree have been used as in traditional Chinese medicine to treat asthma, cough, and enuresis [25]. In 2014, Ambrish Singh and colleagues demonstrated that the fruit extract of *G. biloba* exhibited a potent inhibition of the corrosion of J55 steel in 3.5 wt.% NaCl solution saturated with CO_2_, indicating that the extract formed a protective film on the metal surface and effectively protected J55 steel samples from the corrosive environment [31]. Recently, the fruit extract has been gaining increasing attention because of its potential pharmacological activities and relatively few phytochemical studies. In our recent phytochemical investigations of the fruit extract, we identified one new phenylpropanoid glycoside, namely ginkgopanoside, and we evaluated its 2,2-diphenyl-1-picrylhydrazyl (DPPH) scavenging activities [32]. Additionally, its structure-activity relationships were investigated, which showed that the aglycone of ginkgopanoside displayed more potent activities, indicating that the presence of glucose may decrease the DPPH scavenging activity. Thus, it is meaningful to discover additional new bioactive natural compounds from the extract of *G. biloba* fruits and investigate their potential for pharmacological application.

As part of continuing natural product discovery for bioactive phytochemicals from diverse natural resources [29,33,34,35,36], we investigated natural products from ginkgo fruit (*G. biloba* fruit) with potential protective effects against TNF-α-induced skin aging. Phytochemical investigation of the MeOH extract of *G. biloba* fruits aided by liquid chromatography–mass spectrometry (LC-MS) led to the isolation of 14 compounds (**1**–**14**) from the *n*-butanol (BuOH)-soluble fraction. The structures of the isolated compounds were determined by nuclear magnetic resonance (NMR) spectroscopic data and LC-MS analysis. The anti-skin aging effects of the isolates were examined using TNF-α-stimulated normal human dermal fibroblasts (NHDFs). Herein, we describe the separation and structural elucidation of compounds **1**–**14**, offer an evaluation of their anti-skin aging effects, and the show the underlying molecular mechanisms for the active compound.

## 2. Materials and Methods

### 2.1. General Experimental Procedure

Optical rotation was measured using a Jasco P-2000 polarimeter (Jasco, Easton, MD, USA). Infrared spectra were recorded on a Bruker IFS-66/S FT-IR spectrometer (Bruker, Karlsruhe, Germany). UV spectra were acquired using an Agilent 8453 UV-visible spectrophotometer (Agilent Technologies, Santa Clara, CA, USA). NMR spectra were recorded using a Bruker AVANCE III HD 850 NMR spectrometer with a 5 mm TCI CryoProbe operated at 850 MHz (^1^H) and 212.5 MHz (^13^C). The chemical shifts were presented in ppm (δ) for ^1^H and ^13^C NMR analyses. Preparative and semi-preparative high-pressure liquid chromatography (HPLC) was performed using a Waters 1525 Binary HPLC pump with a Waters 996 photodiode array detector (Waters Corporation, Milford, MA, USA) and Agilent Eclipse C18 column (250 × 21.2 mm, 5 μm; flow rate: 5 mL/min; Agilent Technologies), as well as a Phenomenex Luna Phenyl-hexyl 100 Å column (250 × 10 mm, 5 μm; flow rate: 2 mL/min; Phenomenex, Torrance, CA, USA). LC/MS analysis was performed using an Agilent 1200 Series HPLC system equipped with a diode array detector, a 6130 Series Electrospray Ionizing mass spectrometer for electrospray ionization high-resolution mass spectrometry (ESI-HRMS), and an analytical Kinetex C18 100 Å column (100 × 2.1 mm, 5 μm; flow rate: 0.3 mL/min; Phenomenex). All ESI-HRMS data were obtained using an Agilent G6545B quadrupole time-of-flight mass spectrometer (Agilent Technologies). Silica gel 60 (230–400 mesh; Merck, Darmstadt, Germany) was used for the column chromatography. Diaion HP-20 (Mitsubishi Chemical, Tokyo, Japan) was used for the open-column chromatography. Thin-layer chromatography was performed using pre-coated silica gel F254 plates and RP-C18 F254s plates (Merck), and the spots were detected under UV light or by heating after spraying with anisaldehyde-sulfuric acid.

### 2.2. Plant Material

Whole fruits of *G. biloba* were collected on the campus of Sungkyunkwan University, Suwon, Korea, in October 2019. The plant was identified by one of the authors (K.H.K.). A voucher specimen (GBF-2019-10) was deposited in the herbarium of the School of Pharmacy, Sungkyunkwan University, Suwon, Korea.

### 2.3. Extraction and Separation of Compounds ***1**–**14***

Fresh *G. biloba* fruits (4 kg) were crushed and then extracted twice using 100% MeOH (8.0 L) for 5 days at room temperature, and then the filtrate was concentrated under reduced pressure using a rotary evaporator to obtain the MeOH extract (425.2 g). The MeOH crude extract was suspended in distilled water (700 mL) then partitioned with hexane, dichloromethane (CH_2_Cl_2_), ethyl acetate (EtOAc), and *n*-BuOH three times (700 mL each). The organic phases were evaporated in a vacuum at up to 40 °C to yield the following four soluble fractions: hexane (8.2 g), CH_2_Cl_2_ (1.9 g), EtOAc (4.0 g), *n*-BuOH (28.8 g). With reference to a house-built UV library, the *n*-BuOH-soluble fraction presented the majority of phenolic compounds based on LC/MS analysis of the four fractions. One part of the *n*-BuOH-soluble fraction (GSB, 2.0 g), the portion without sugar, obtained by Diaion HP-20 column, was chromatographed on a silica gel column eluting with CH_2_Cl_2_/MeOH step gradients to yield nine fractions (GSB1–GSB9) obtained by combining the eluates based on TLC analysis. Fraction GSB6 (366.4 mg) was subjected by preparative reversed phase HPLC (prep. RP-HPLC) and eluted with a gradient solvent system of MeOH/H_2_O (40–100% MeOH) at a flow rate of 5 mL/min to afford three fractions (GSB61–GSB63). Fraction GSB62 (189.1 mg) was then subjected to column chromatography on silica gel eluted with CH_2_Cl_2_/MeOH step gradients to give six subfractions (GSB62a–GSB62f). GSB62a (88.3 mg) was further purified using semi-preparative reversed phase HPLC (semi-prep. RP-HPLC) with an isocratic solvent system of 27% MeOH/H_2_O at a flow rate of 2 mL/min to obtain compounds 1 (2.3 mg, purity = 91%) and 2 (1.4 mg, purity = 87%). Similarly, GSB62c (46.0 mg) was isolated using semi-prep. RP-HPLC with 30% MeOH/H_2_O to yield compounds 3 (3.2 mg, purity = 95%), 4 (2.2 mg, purity = 94%), 5 (1.1 mg, purity = 86%), 6 (0.8 mg, purity = 90%) and 7 (1.0 mg, purity = 92%). Fraction GSB7 (257.0 mg) was subjected to column chromatography on silica gel eluted with CH_2_Cl_2_/MeOH step gradients to obtain ten subfractions (GSB71–GSB79). Compounds 8 (6.7 mg, purity = 91%) and 9 (5.5 mg, purity = 92%) were separated from GSB73 (58.9 mg) using semi-prep. RP-HPLC with an isocratic solvent system of 15% MeOH/H_2_O at a flow rate of 2 mL/min. GSB79 (99.7 mg) was further fractionated by prep. RP-HPLC using a gradient solvent system of MeOH/H_2_O (30–80% MeOH, flow rate of 5 mL/min) to obtain four subfractions (GSB79a–GSB79d). Successfully, compounds 10 (1.4 mg, purity = 88%) and 11 (2.3 mg, purity = 91%) were yielded from GSB79b using the same semi-prep. RP-HPLC system with 18% MeOH/H_2_O. Fraction GSB8 (258.7 mg) was performed to prep. RP-HPLC and eluted with a gradient solvent system of MeOH/H_2_O (30–80% MeOH) at a flow rate of 5 mL/min to afford four fractions (GSB81–GSB84). Of these, GSB81 was further submitted to Sephadex LH-20 column chromatography and obtained four subfractions (GSB81a–GSB81d). GSB81b (67.1 mg) was further purified by semi-prep. RP-HPLC with 35% MeOH/H_2_O to afford compound 12 (11.3 mg, purity = 90%). Lastly, compounds 13 (1.3 mg, purity = 88%) and 14 (0.9 mg, purity = 90%) were isolated from GSB81d (43.6 mg) using semi-prep. RP-HPLC with 35% MeOH/H_2_O.

### 2.4. Cell Culture and Treatment Preparation

NHDFs (juvenile foreskin, cryopreserved, 5 × 10^5^ cells) were obtained from PromoCell (Sickingenstr, Heidelberg, Germany) and stored in a nitrogen tank. NHDFs were cultured in Dulbecco’s modified Eagle’s medium (DMEM; Corning, Manassas, VA, USA, pH 7.4) containing 10% fetal bovine serum (FBS; Atlas, Fort Collins, CO, USA) and 100 U/mL penicillin–streptomycin (Gibco, Grand Island, NY, USA) in a humidified atmosphere containing 5% CO_2_ at 37 °C in a cell incubator (Thermo Scientific, Waltham, MA, USA). The isolated compounds were dissolved in dimethyl sulfoxide (Biosesang, Seong-Nam, Korea) to prepare 10 mM stock solutions. The isolated compounds were diluted in DMEM without FBS at concentrations of 25, 50, and 100 μM. The concentration of the TNF-α stock solution was 20 μg/mL, and the final treatment concentration was 20 ng/mL. In all experiments, the cells were seeded onto a cell culture plate and starved in DMEM without FBS overnight. The cells were then co-treated with TNF-α and the isolated compounds.

### 2.5. Cell Viability

NHDFs were seeded in 96-well plates with a clear bottom (5 × 10^3^ cells/well) and incubated for 24 h. The NHDF were then starved in DMEM without FBS for 24 h. The supernatant was removed and replaced with a serum-free medium. The isolated compounds were exposed to specified concentrations and incubated for 24 h in a cell incubator. Next, 100 μL EZ-Cytox solution (Dogen, Seoul, Korea) was added to each well and incubated for 1 h. The absorbance values were measured using a plate reader (SPARK 10 M; Tecan) at 450 nm.

### 2.6. Intercellular ROS Generation Assay

NHDFs were plated in black 96-well plates with a flat bottom (1 × 10^4^ cells/well) and incubated for 24 h in a cell incubator. To arrest the cell cycle, cells were stored in DMEM without FBS for 24 h. After this process, the NHDFs were treated with 25, 50, and 100 µM of the isolated compounds for 1 h and co-treated with specific concentrations of TNF-α (20 ng/mL) and DCFDA (10 µM) (Sigma-Aldrich, St. Louis, MO, USA; CAT No. 35845-1G) for 15 min. After aspiration of the supernatant, phosphate-buffered saline (PBS, pH 7.4) (Welgene, Gyeongsangbuk, Korea) was added to each well for washing. The level of intercellular ROS generation was measured by DCFDA treatment using a microplate reader (SPARK 10 M) at wavelengths of 485 and 535 nm. For fluorescence imaging, NHDFs were seeded in 48-well clear plate (1 × 10^4^ cells/well) and incubated for 24 h in a cell incubator. Subsequently, the next process was performed as described above. DCFDA-stained images were immediately observed using a fluorescence microscope (Olympus, Tokyo, Japan).

### 2.7. Enzyme-Linked Immunosorbent Assay (ELISA)

NHDFs were seeded in 48-well plates at a flat bottom (2 × 10^4^ cells/well) and incubated for 24 h. The cells were then starved in a serum-free medium for 24 h. L3R was then added at the indicated concentrations for 1 h. Subsequently, cells were treated with 20 ng/mL TNF-α for 24 h. The secretion of MMP-1 and COLIA1 was measured using ELISA kits (R&D systems, Minneapolis, USA; MMP-1 kit, CAT NO. DY091B; COLIA1 kit, CAT NO. DY6220-05).

### 2.8. Western Blotting

NHDFs were seeded in 6-well plates at a flat bottom (3 × 10^5^ cells/well) and incubated for 24 h. The NHDF were then starved in serum-free medium for 24 h. The cells were treated with L3R at the indicated concentration for 1 h. The cells were then treated with 20 ng/mL TNF-α for 15 min (for protein expression analysis of ERK, p-ERK, p38, p-p38, JNK, and p-JNK). Each well was then washed with PBS and lysed using radioimmunoprecipitation buffer (pH 8.0). After collection, the samples were centrifuged at 13,000 rpm for 30 min and harvested for western blot analysis. The protein concentrations of the harvested supernatants were detected using a BCA protein assay kit (Merck, Darmstadt, Germany). Standardized amounts of samples were loaded onto a 4–20% precast polyacrylamide gel (Bio-Rad, Hercules, CA, USA) and transferred to polyvinylidene difluoride membranes (PVDF) (Merck, Darmstadt, Germany). The membranes were blocked with 5% non-fat dry milk (BD Difco, CAT No. 232100) in Tris-buffered saline (pH 7.4) containing 0.1% Tween-20 (TBS-T; Fisher Scientific, Fair Lawn, NJ, USA; CAT No. BP337-500) for 24 h. The membranes were incubated with primary antibodies (Cell Signaling Technology, Danvers, MA, USA) diluted in 1% BSA (ERK, p-ERK, JNK, p-JNK, p38, p-p38, and GAPDH) overnight at 4 °C and washed three times with TBS-T. The membranes were then incubated with secondary rabbit antibodies (Cell Signaling Technology) at room temperature for 2 h and washed with TBS-T thrice. Finally, the membranes were treated with SuperSignal Femto Substrate reagent kit (Thermo Scientific, Waltham, MA, USA). Protein bands were analyzed using the Fusion Solo Chemiluminescence System (PEQLAB Biotechnologie GmbH, Erlangen, Germany).

### 2.9. Statistical Analyses

All results are presented as mean ± standard error of the mean of at least two independent experiments. Statistical analyses were carried out using one-way analysis of variance (ANOVA) followed by Tukey’s honest significant difference test. Statistical significance was set at *p* < 0.05.

## 3. Results

### 3.1. Isolation and Structure Elucidation of Compounds

**Figure 1:**
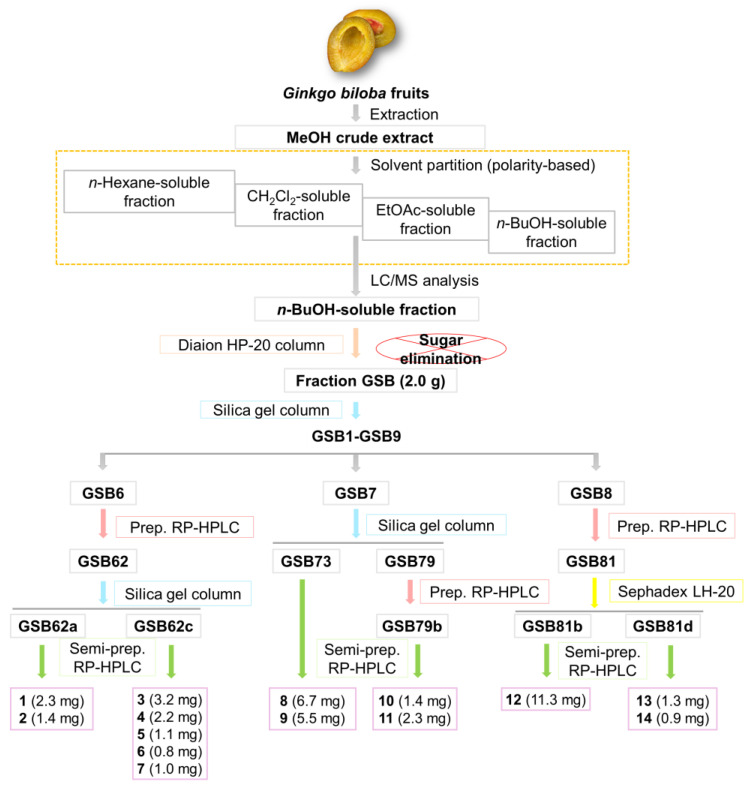
The separation scheme of compounds **1**–**14**.

**Figure 2:**
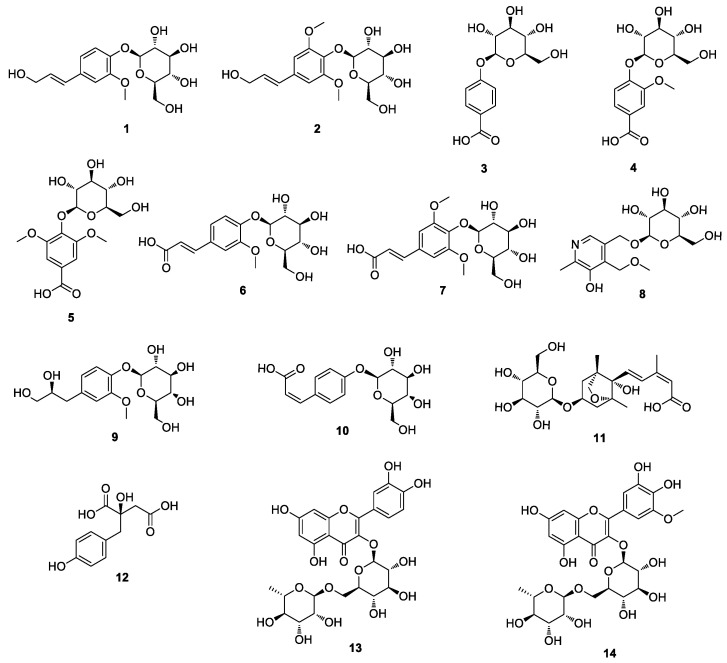
Structures of compounds **1**–**14** isolated from *G. biloba* fruits.

G. biloba fruits were extracted with 100% MeOH at room temperature and the resultant MeOH extract was successively partitioned with four representative organic solvents to obtain four main fractions: hexane, CH_2_Cl_2_, EtOAc, and n-BuOH-soluble fractions (Figure 1). The analysis by LC-MS using the house-built UV library database revealed that the n-BuOH-soluble fraction was rich in phenolic compounds, including flavonoids. The column chromatographic procedures including semi-preparative HPLC separation led to the isolation of 14 compounds from the n-BuOH-soluble fraction (Figure 1). The isolated compounds were structurally determined to be: (E)-coniferin (**1**) [37]; syringin (**2**) [38]; 4-hydroxybenzoic acid 4-O-β-D-glucopyranoside (**3**) [39]; vanillic acid 4-O-β-D-glucopyranoside (**4**) [40]; glucosyringic acid (**5**) [41]; (E)-ferulic acid 4-O-β-D-glucoside (**6**) [42]; (E)-sinapic acid 4-O-β-D-glucopyranoside (**7**) [43]; ginkgotoxin-5-glucoside (**8**) [44]; ginkgopanoside (**9**) [32]; (Z)-4-coumaric acid 4-O-β-D-glucopyranoside (**10**) [45]; (1′R,2′S,5′R,8′S,2′Z,4′E)-dihydrophaseic acid 3′-O-β-D-glucopyranoside (**11**) [46]; eucomic acid (**12**) [47]; rutin (**13**) [48]; and laricitrin 3-rutinoside (L3R) (**14**) [49] (Figure 2). These determinations were made by spectral analysis (Figures S1-S28), mainly LC/MS, UV, and NMR experiments, and comparison of their spectroscopic data with those previously reported.

### 3.2. Effect of Compounds ***1**–**14*** on NHDFs Viability

**Figure 3:**
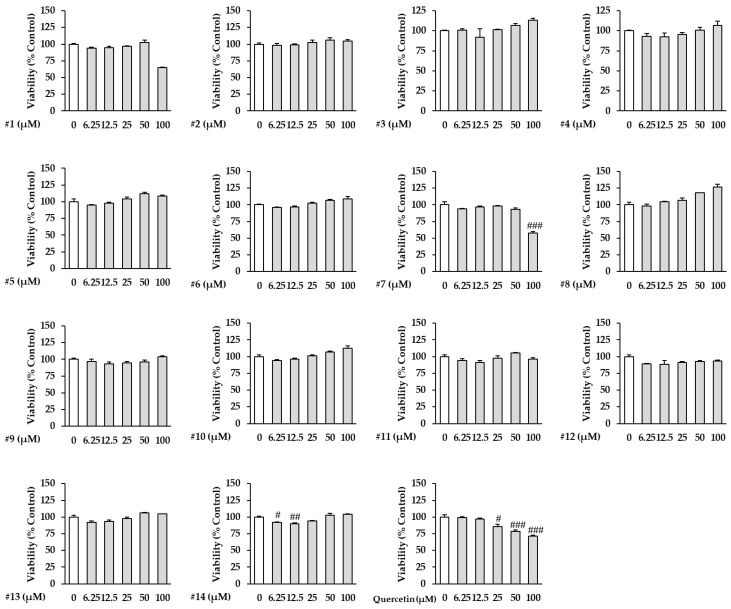
Effect of the isolated compounds **1**–**14** on NHDF viability. NHDFs were seeded in 96-well cell culture plates at a clear bottom (5 × 10^3^ cells/well) for 24 h and starved in fresh DMEM without surum for 24 h. The cells were then treated with the indicated concentrations of compounds **1**–**14** for 24 h. Cell viabillty by compounds **1**–**14** was evaluated using an Ez-Cytox kit. The results are presented as the mean ± standard error of the mean (SEM) (n = 2). # *p* < 0.05, ## *p* < 0.01, and ### *p* < 0.001 compared to the untreated group.

In this study, we evaluated the effect of the isolated compounds **1**–**14** against TNF-α-induced damage to NHDFs. Before evaluating this effect, the viability of NHDFs treated with the isolated compounds **1**–**14** was assessed. The NHDFs were treated with compounds **1**–**14** at specific concentrations to evaluate cell viability, and it was found that the cell viability did not decrease up to 100 µM, except for compounds **1** and **7** (Figure 3). On the other hand, the extract of G. biloba fruits did not decrease the cell viability up to 12.5 μg/mL (Figure S29).

### 3.3. Effect of Compounds ***1**–**14*** on Intracellular ROS Generation in TNF-α-Stimulated NHDFs

**Figure 4:**
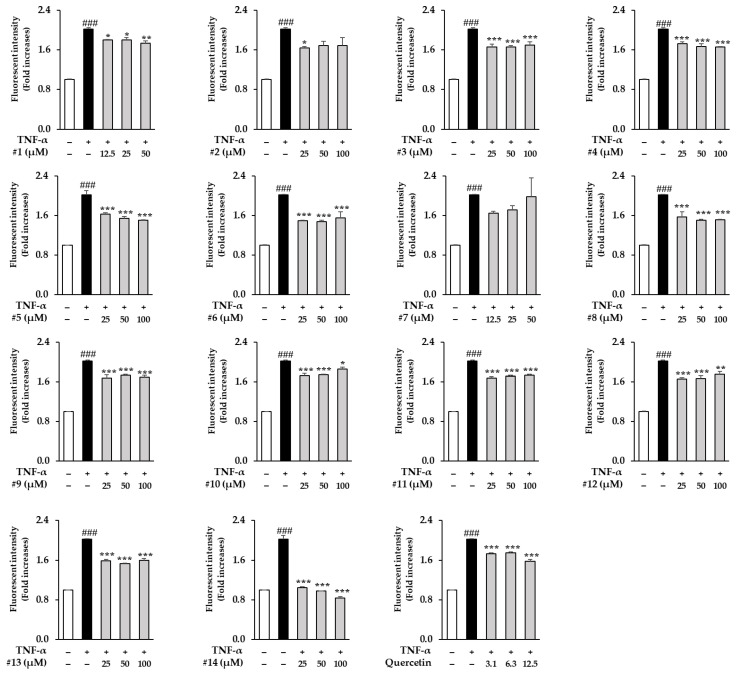
Effects of isolated compounds **1**–**14** on TNF-α-induced intercellular reactive oxygen species (ROS) generation. NHDFs were plated in a 96-well black plate with a clear flat bottom, containing 1 × 10^4^ cells per well, and incubated for 24 h. The cells were stored under starved conditions for 24 h. Next, the cells were pretreated with specific concentrations of the isolated compounds **1**–**14** for 1 h and exposed to specific concentrations of TNF-α (20ng/mL) and DCFDA (10μM) for 15 min. ROS generation was measured using a microplate reader. Data are shown as the mean ± S.E.M. of duplicate experiments. ### *p* < 0.001 versus the untreated value,* *p* < 0.05, ** *p* < 0.01, and *** *p* < 0.001 versus the TNF-α-treated value.

The TNF-α receptor on the cell surface is activated by repetitive UV exposure of the skin [50]. As a result, increased TNF-α levels provoke inflammatory responses that cause oxidative stress, which then stimulates intracellular ROS generation in NHDFs [51]. Therefore, we assessed the effects of the isolated compounds **1**–**14** on intracellular ROS generation in TNF-α-stimulated NHDFs. Compared to the control group, treatment with 20 ng/mL TNF-α increased ROS generation by 2.02 ± 0.07-fold (*p* < 0.001). As a result, the compound **6**-treated group inhibited ROS generation to 1.50 ± 0.01 (*p* < 0.001 at 25 μM), 1.48 ± 0.02 (*p* < 0.001 at 50 μM), and 1.55 ± 0.12 (*p* < 0.001 at 100 μM) when compared to the TNF-α group (2.02 ± 0.00-fold, *p* < 0.001) (Figure 4). On the contrary, the compound **14** (L3R)-treated group remarkably reduced the ROS generation to 1.05 ± 0.01 (*p* < 0.001 at 25 μM), 0.98 ± 0.00 (*p* < 0.001 at 50 μM), and 0.84 ± 0.03 (*p* < 0.001 at 100 μM) when compared to the TNF-α group (2.02 ± 0.07-fold, *p* < 0.001). As a result of the positive control, quercetin reduced ROS generation to 1.73 ± 0.01 (*p* < 0.001 at 3.1 μM), 1.75 ± 0.01 (*p* < 0.001 at 6.3 μM), and 1.58 ± 0.02 (*p* < 0.001 at 12.5 μM) when compared to the TNF-α group (2.02 ± 0.01-fold, *p* < 0.001). As mentioned above, L3R inhibited TNF-α-induced intracellular ROS generation in NHDFs. Thus, we conducted further studies to evaluate the protective effects of L3R against TNF-α-induced damage in NHDFs. On the other hand, the extract of G. biloba fruits also supressed ROS generation at 1.5-6.25 μg/mL when compared to the TNF-α group (Figure S30).

### 3.4. Effect of L3R on ROS Generation in TNF-α-Treated NHDFs

**Figure 5:**
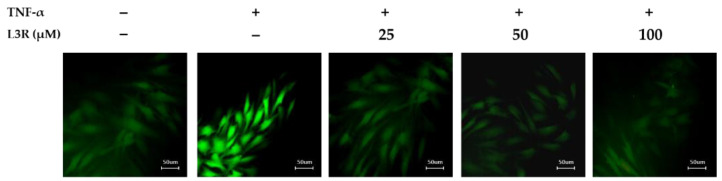
Effects of L3R on TNF-α-induced intercellular reactive oxygen species (ROS) generation. NHDFs were seeded in a 48-well plate with a clear flat bottom (2 × 10^4^ cells/well) and were incubated for 24 h. The cells were stored under starved conditions for 24 h. Next, cells were pretreated with the indicated concentrations of L3R for 1 h and exposed to specific concentration of TNF-α (20 ng/mL) and DCFDA (10 μM) for 15 min. Subsequently, the DCFDA stainded cells were captured by fluorescence microscopy. Scal bars indicate 50 μm.

To observe the effects of L3R on TNF-α-induced intercellular ROS generation, we conducted a DCFDA staining assay. As depicted in Figure 5, L3R exhibited an inhibitory effect on ROS generation in the TNF-α-treated groups. These findings further supported the results of L3R obtained from the intracellular ROS generation assay, as shown in Figure 4.

### 3.5. Effect of L3R on MMP-1 and COLIA1 Protein Secretion in TNF-α-Treated HDFs

**Figure 6:**
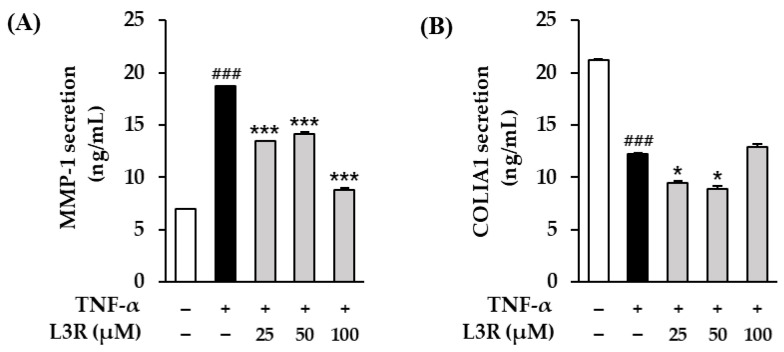
Effect of laricitrin 3-rutinoside (L3R) on protien secretion (MMP-1 and COLIA1; Type 1 collagen alpha 1) in tumor necrosis factor-α (TNF-α)-stimulated normal human dermal fibroblasts (NHDFs). NHDFs were treated with 25, 50, or 100 µM L3R for 1 h, followed by treatment with 20 ng/mL TNF-α for 24 h. MMP-1 (**A**) and COLIA1 (**B**) levels were measured using an ELISA kits. Data are shown as mean ± S.E.M. of triplicate experiments. ### *p* < 0.001 versus the control value; * *p* < 0.05, and *** *p* < 0.001 versus TNF-α-treated values.

Recall that TNF-α increases intracellular ROS, which stimulates the expression of collagenases, including MMP-1 and MMP-9 [52]. These collagenases destroy the ECM and cause skin aging [53]. Therefore, we evaluated the effects of L3R on MMP-1 and COLIA secretion in TNF-α-treated NHDFs (Figure 6). In comparison to the control group, treatment with 20 ng/mL TNF-α significantly increased MMP-1 secretion to 18.7 ± 0.11 ng/mL. In contrast, treatment with 25, 50, and 100 µM of L3R diminished MMP-1 secretion to 13.5 ± 0.01 (*p* < 0.001), 14.1 ± 0.36 (*p* < 0.001), and 8.80 ± 0.37 ng/mL (*p* < 0.001), respectively. The TNF-α-treated group significantly reduced COLIA1 secretion to 12.3 ± 0.10 ng/mL (*p* < 0.001). In contrast, treatment with 100 µM of L3R increased COLIA1 secretion to 12.9 ±. 0.50 ng/mL (*p* < 0.01). We also performed MMP-1 and COLIA1 assays on the extract of G. biloba fruits.. The extract supressed MMP-1 secretion at 3.1-6.25 μg/mL when compared to the TNF-α group, and increased COLIA1 secretion at 1.5-6.25 μg/mL when compared to the TNF-α group (Figure S31).

### 3.6. Effect of L3R on MAPKs Phosphorylation in TNF-α-Treated HDFs

**Figure 7:**
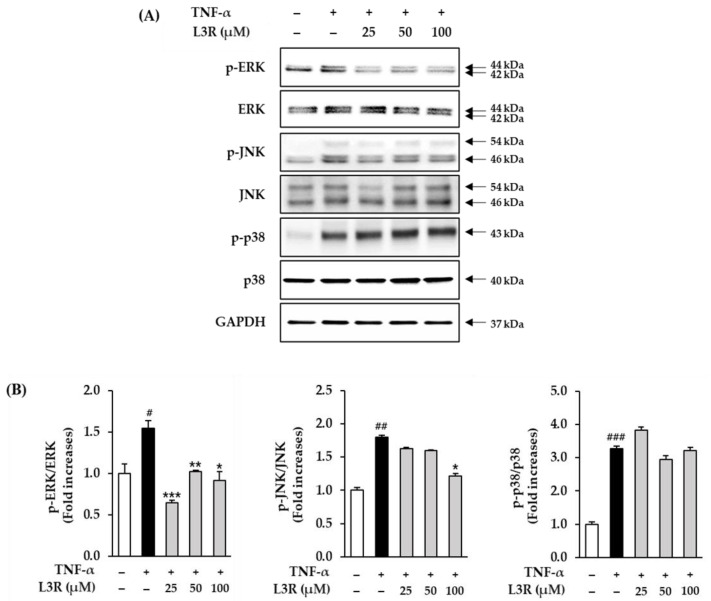
Effect of L3R on TNF-α-induced protein expression associated with inhibition of mitogen-activated protein kinases (MAPKs) phosphorylation in normal human dermal fibroblasts (NHDFs). (**A**) Cells were treated with 25, 50, and 100 µM L3R for 1 h and then exposed to 20 ng/mL TNF-α for 15 min. The phosphorylated bands were analyzed by immunoblotting for phospho-ERK, ERK, phospho-JNK, JNK, phospho -p38, p38, and GAPDH. (**B**) The graphs show the fold-increase in the phosphorylation of MAPKs compared to the untreated group. Data are presented as mean ± SEM (n = 3). # *p* < 0.05, ## *p* < 0.01, and ### *p* < 0.001 compared with the control group and * *p* < 0.05, ** *p* < 0.01, and *** *p* < 0.001 compared with the TNF-α-treated group.

TNF-α-induced ROS promotes the phosphorylation of MAPKs, which in turn regulate the expression of collagenase and pro-inflammatory cytokines [54]. Therefore, we evaluated the effect of L3R on MAPKs phosphorylation in TNF-α-exposed NHDFs. It was found that treatment with 20 ng/mL of TNF-α promoted ERK phoypholyation to 1.13 ± 0.01 (*p* < 0.05) when compared to the control group (Figure 7). In contrast, the indicated concentration of L3R significantly inhibited ERK phoypholyation to 0.50 ± 0.02 (*p* < 0.001, at 25 µM), 0.75 ± 0.02 (*p* < 0.001, at 50 µM), and 0.73 ± 0.02 (*p* < 0.001, at 100 µM). In addition, TNF-α promoted JNK phoypholyation 1.80 ± 0.04 (*p* < 0.01) while 100 µM of L3R treatment decreased it by 1.22 ± 0.06 (*p* < 0.05) (Figure 7).

### 3.7. Effect of L3R on Pro-Inflammatory Cytokine Secretion in TNF-α-Treated HDFs

**Figure 8:**
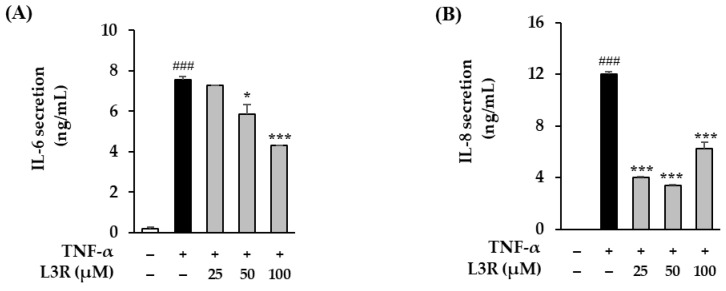
Effect of L3R on IL-6 and IL-8 protein expression in TNF-α-induced NHDFs. NHDFs were treated with 25, 50, or 100 µM L3R for 1 h, followed by treatment with 20 ng/mL TNF-α for 24 h. IL-6 (**A**) and IL-8 (**B**) levels were measured using ELISA kits. Data are shown as mean ± S.E.M. of duplicate experiments. ### *p* < 0.001 versus the control value; * *p* < 0.05, and *** *p* < 0.001 versus the TNF-α-treated value.

Pro-inflammatory cytokines cause chronic diseases, such as psoriasis, that destroy alpha hydroxy acids in the ECM and acclerate skin aging [55]. Therefore, we evaluated the effects of L3R on IL-6 and IL-8 secretion in TNF-α-treated NHDFs. Compared to the control group, a treatment of 20 ng/mL TNF-α increased IL-6 secretion to 7.57 ± 0.14 ng/mL (*p* < 0.001). In contrast, 50 and 100 µM of L3R treatment decreased it to 5.88 ± 0.47 (*p* < 0.05) and 4.32 ± 0.00 (*p* < 0.001), respectively (Figure 8). In IL-8 secretion, TNF-α treatment significantly increased IL-8 secertion to 12.04 ± 0.19 ng/mL (*p* < 0.001). However, 25, 50, and 100 µM of L3R treatment attenuated it to 4.03 ± 0.03 (*p* < 0.001), 3.38 ± 0.06 (*p* < 0.001), and 6.23 ± 0.49 ng/mL (*p* < 0.001), respectively.

## 4. Discussion

The human skin is the primary barrier against harmful external factors such as bacterial fungi and chemical UV radiation [56]. The skin comprises two main functional layers: epidermal and dermal. The two layers interact with each other. The epidermis is a thin layer that easily recovers after injury and maintains moisture inside the body. However, it does not have a vascular layer, which should be supported by another assistant. The dermis is a thick layer of blood vessels, collagen, and elastin fibers [57]. It transports nutrients to the epidermis to maintain the moisture content [58]. Skin aging can be divided into two categories: endogenous and exogenous. Endogenous aging factors are generated within the human body, such as stress, genetic constitution of an individual person, and disease [59,60]. Exogenous factors, such as UV radiation, smoking, and pollution, are generated outside the body. Both aging factors decrease collagen and elastin fibers and fibroblasts [61]. In particular, UV radiation can directly destroy dermal collagen [62]. UV radiation induces various skin aging factors such as TNF-α. TNF-α causes inflammatory reactions in the skin that promote oxidative stress. An increase in oxidative stress is accompanied by ROS generation [63,64]. Excessive ROS generation promotes photoaging, which destroys the dermal layer and leads to epidermal damage. Consequently, skin aging occurs, and symptoms appear in human skin, such as wrinkles, pigments, and sagging [65,66].

As previously mentioned, inhibition of TNF-α-induced ROS is the key to protecting the skin against photoaging and skin disease. Therefore, we investigated potential natural products with protective effects against TNF-α-induced skin aging from *G. biloba* fruit. Phytochemical investigation of the MeOH extract of *G. biloba* fruits led to the isolation of compounds **1**–**14**; (*E*)-coniferin (**1**), syringin (**2**), 4-hydroxybenzoic acid 4-O-β-D-glucopyranoside (**3**), vanillic acid 4-O-β-D-glucopyranoside (**4**), glucosyringic acid (**5**), (*E*)-ferulic acid 4-O-β-D-glucoside (**6**), (*E*)-sinapic acid 4-O-β-D-glucopyranoside (**7**), ginkgotoxin-5-glucoside (**8**), ginkgopanoside (**9**), (*Z*)-4-coumaric acid 4-O-β-D-glucopyranoside (**10**), (1′*R*,2′*S*,5′*R*,8′*S*,2′*Z*,4′*E*)-dihydrophaseic acid 3′-O-β-D-glucopyranoside (**11**), eucomic acid (**12**); rutin (**13**), and L3R (**14**). According to the literature survey, compounds **3-5** were isolated from *G. biloba* for the first time in this study. Among the isolated compounds, L3R (**14**) significantly inhibited intracellular ROS generation in TNF-α-treated NHDFs. However, the protective effects of L3R on TNF-α-induced NHDFs have not yet been reported. Thus, several assays should be conducted to identify the signaling pathway for the ROS-related downregulation of L3R in TNF-α-induced skin aging.

Furthermore, TNF-α-induced ROS generation is known to decrease collagen synthesis and increase the synthesis of collagenases, including MMPs [67]. TNF-α increased the expression of MMP-1, matrix metalloproteinase-3 (MMP-3), and matrix metalloproteinase-9 (MMP-9). Among these MMPs, MMP-1, which initiates collagen cleavage, is a crucial factor in skin aging [68]. In addition, an increase in collagenase expression is associated with type I collagenolysis [69]. Thus, we evaluated the inhibitory effect of L3R on MMP-1 secretion and destruction of type 1 collagen in TNF-α-exposed NHDFs. Figure 6 shows that L3R diminishes MMP-1 secretion and type 1 collagen degradation. Similar to our findings, previous studies have demonstrated that flavonoids derived from natural sources exhibit anti-skin aging activity. For instance, potentilloside A has been shown to inhibit ROS generation and MMP-1 secretion in TNF-α-induced HDFs [70]. Additionally, quercetin, myricetin, and kaempferol have been reported to inhibit MMP-1 expression and superoxide anion radicals in UV-induced human dermal fibroblasts [71]. Furthermore, 6-hydroxyflavones have been found to increase collagen type 1 mRNA expression in normal human skin fibroblasts [72].

TNF-α-induced ROS generation are known to upregulate skin aging-related molecular pathways such as MAPKs and NF-κB, while ROS trigger the activation of MAPKs phosphorylation, which in turn upregulates the transcriptional pathways NF-κB and AP-1 [73,74]. In a previous study, MAPKs were found to upregulate NF-kB and AP-1, which increased the expression of MMP-1 and pro-inflammatory cytokines such as IL-6, IL-8, and IL-1. Therefore, we investigated the inhibitory effect of L3R on MAPK phosphorylation in TNF-α-induced NHDFs. Figure 7 shows that L3R inhibits ERK phosphorylation in TNF-α-stimulated NHDFs. Similar to our results, several studies have demonstrated that flavonoids can effectively suppress the generation of ROS and inhibit MMP-1 production by targeting key signaling pathways such as MAPKs and NF-κB in human dermal fibroblasts and keratinocytes. For instance, apigenin and luteolin have been found to suppress UVA-induced matrix metalloproteinase-1 expression through MAPKs- and AP-1-dependent signaling in HaCaT cells [75]. Additionally, Skullcapflavone II has been shown to inhibit the degradation of type I collagen by suppressing MMP-1 transcription via ERK, JNK/AP-1, and NF-κB in TNF-α-induced human dermal fibroblasts [76].

As pro-inflammatory factors cause diverse inflammatory reactions that lead to chronic diseases [77], we investigated the effect of L3R on pro-inflammatory cytokine secretion in TNF-α-stimulated NHDFs. Figure 8 indicates that L3R has an inhibitory effect on proinflammatory factors in TNF-α-induced NHDFs. Flavonoids have been widely reported to exhibit suppressive effects on pro-inflammatory cytokines through the modulation of MAPKs/AP-1 or NF-κB signaling pathways in human skin cells. For instance, catechin has been shown to inhibit ROS generation and reduce levels of MMP-1 and pro-inflammatory cytokines. Furthermore, catechin has been found to increase the secretion of pro-collagen type 1 through MAPKs/AP-1 and NF-κB pathways in TNF-α-induced HDFs [78]. Similarly, fisetin has been demonstrated to ameliorate ROS generation, MMPs (MMP-1, 3, 9), and pro-inflammatory cytokines through the inhibition of MAPKs and NF-κB signaling in UVB-induced human foreskin fibroblasts (HS68) [79]. The aforementioned studies provide support for the hypothesis that L3R can effectively ameliorate ROS generation and inhibit MMP-1 secretion through the suppression of ERK phosphorylation.

**Figure 9:**
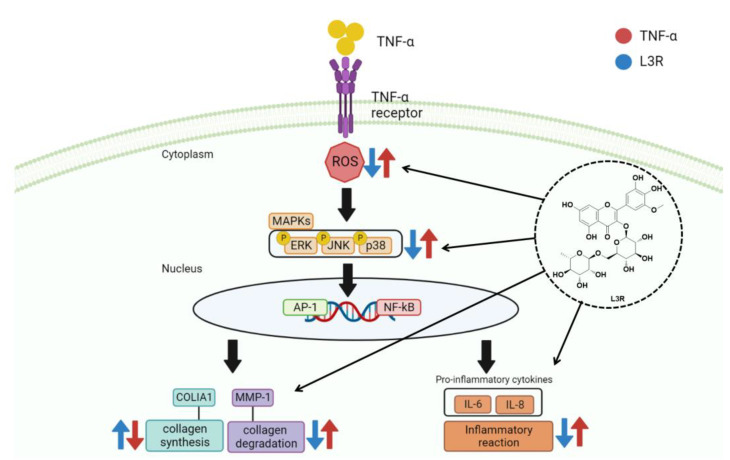
Schematic illustration of the potential protective effect of L3R in TNF-α-induced NHDFs.

These results suggest that L3R is a potential protective agent against the TNF-α-induced skin aging process, and we have summarized the mechanism of the protective effect of L3R against TNF-α-induced NHDFs in Figure 9. In addition to the protective effect of L3R on TNF-α-induced skin aging, there are other potential effects associated with L3R. For instance, flavonoids have been found to inhibit fibrosis both in vitro and in vivo. Luteolin, quercetin, and myricetin have shown a dose-dependent inhibition of Smad2/3 phosphorylation in human dermal fibroblasts [80]. Additionally, quercetin has been shown to inhibit kidney fibrosis by reducing αSMA and fibronectin expression in mice with unilateral ureteral obstruction (UUO) [81]. Furthermore, rutin (quercetin 3-rutinoside) has demonstrated a decrease in α-SMA and collagen expression by inhibiting TGF-β/Smad signaling in BDL-induced lung tissue of Sprague–Dawley rats (SD rats) [82]. Therefore, L3R not only holds potential as a candidate for addressing skin aging but also shows potential application in other conditions, including fibrosis.

## 5. Conclusions

In conclusion, in an ongoing study focused on the discovery of potential bioactive phytochemicals, we investigated protective natural products from ginkgo fruit (*G. biloba* fruit) against skin aging by TNF-α stimulation. Phytochemical examination of the MeOH extract of *G. biloba* fruit resulted in the isolation of 14 compounds (1–14) using column chromatography and HPLC purification aided by LC-MS analysis. Among the isolated compounds, L3R (14) was found to exhibit protective activity by inhibiting TNF-α-induced ROS generation in NHDFs. L3R also diminishes collagenase secretion and prevents collagen destruction by suppressing ERK phosphorylation. Furthermore, L3R inhibits pro-inflammatory cytokines, such as IL-6 and IL-8, which stimulate diverse inflammatory reactions associated with skin aging. Although further studies are needed, these results suggest that L3R is a potential protective agent against TNF-α-induced skin aging.

## Data Availability

Not applicable.

## Supplementary Materials

The following are available online at https://www.mdpi.com/article/10.3390/antiox12071432/s1: Figure S1: ^1^H-NMR (CD_3_OD, 850 MHz) spectrum of (*E*)-coniferin (**1**); Figure S2: UV chromatogram of LC/MS (A: monitored at 254 nm) and UV (B) and MS data (C: positive; D: negative) for **1**. Figure S3: ^1^H-NMR (CD_3_OD, 850 MHz) spectrum of syringin (**2**); Figure S4: UV chromatogram of LC/MS (A: monitored at 254 nm) and UV (B) and MS data (C: positive; D: negative) for **2**; Figure S5: ^1^H-NMR (CD_3_OD, 850 MHz) spectrum of 4-hydroxybenzoic acid 4-*O*-β-D-glucopyranoside (**3**); Figure S6: UV chromatogram of LC/MS (A: monitored at 254 nm) and UV (B) and MS data (C: positive; D: negative) for **3**; Figure S7: ^1^H-NMR (CD_3_OD, 850 MHz) spectrum of vanillic acid 4-*O*-β-D-glucopyranoside (**4**); Figure S8: UV chromatogram of LC/MS (A: monitored at 315 nm) and UV (B) and MS data (C: positive; D: negative) for **4**; Figure S9: ^1^H-NMR (CD_3_OD, 850 MHz) spectrum of glucosyringic acid (**5**); Figure S10: UV chromatogram of LC/MS (A: monitored at 315 nm) and UV (B) and MS data (C: positive; D: negative) for **5**; Figure S11: ^1^H-NMR (CD_3_OD, 850 MHz) spectrum of (*E*)-ferulic acid 4-*O*-β-D-glucoside (**6**); Figure S12: UV chromatogram of LC/MS (A: monitored at 315 nm) and UV (B) and MS data (C: positive; D: negative) for **6**; Figure S13: ^1^H-NMR (CD_3_OD, 850 MHz) spectrum of (*E*)-sinapic acid 4-*O*-β-D-glucopyranoside (**7**); Figure S14: UV chromatogram of LC/MS (A: monitored at 315 nm) and UV (B) and MS data (C: positive; D: negative) for **7**; Figure S15: ^1^H-NMR (CD_3_OD, 850 MHz) spectrum of ginkgotoxin-5-glucoside (**8**); Figure S16: UV chromatogram of LC/MS (A: monitored at 315 nm) and UV (B) and MS data (C: positive; D: negative) for **8**; Figure S17: ^1^H-NMR (CD_3_OD, 850 MHz) spectrum of ginkgopanoside (**9**); Figure S18: UV chromatogram of LC/MS (A: monitored at 254 nm) and UV (B) and MS data (C: positive; D: negative) for **9**; Figure S19: ^1^H-NMR (CD_3_OD, 850 MHz) spectrum of (*Z*)-4-coumaric acid 4-*O*-β-D-glucopyranoside (**10**); Figure S20: UV chromatogram of LC/MS (A: monitored at 254 nm) and UV (B) and MS data (C: positive; D: negative) for **10**; Figure S21: ^1^H-NMR (CD_3_OD, 850 MHz) spectrum of (1′*R*,2′*S*,5′*R*,8′*S*,2′*Z*,4′*E*)-dihydrophaseic acid 3’-*O*-β-D-glucopyranoside (**11**); Figure S22: UV chromatogram of LC/MS (A: monitored at 254 nm) and UV (B) and MS data (C: positive; D: negative) for **11**; Figure S23: ^1^H-NMR (CD_3_OD, 850 MHz) spectrum of eucomic acid (**12**); Figure S24: UV chromatogram of LC/MS (A: monitored at 254 nm) and UV (B) and MS data (C: positive; D: negative) for **12**; Figure S25: ^1^H-NMR (CD_3_OD, 850 MHz) spectrum of rutin (**13**); Figure S26: UV chromatogram of LC/MS (A: monitored at 254 nm) and UV (B) and MS data (C: positive; D: negative) for **13**; Figure S27: ^1^H-NMR (CD_3_OD, 850 MHz) spectrum of laricitrin 3-rutinoside (**14**); Figure S28: UV chromatogram of LC/MS (A: monitored at 254 nm) and UV (B) and MS data (C: positive; D: negative) for **14**; Figure S29: Effect of the extract on NHDF viability; Figure S30: Effects of the extract on TNF-α-induced intercellular reactive oxygen species (ROS) generation; Figure S31: Effect of the extract on MMP-1 and COLIA1 protein expression in tumor necrosis factor-α (TNF-α)-stimulated normal human dermal fibroblasts (NHDFs).
