# Supplementary material for: Laricitrin 3-Rutinoside from Ginkgo biloba Fruits Prevents Damage in TNF-α-Stimulated Normal Human Dermal Fibroblasts
Source: Antioxidants (Basel). 2023 Jul 15;12(7):1432. doi: 10.3390/antiox12071432 (PMC10376084; doi:10.3390/antiox12071432)
Supplement: Supplementary file 1 [file antioxidants-12-01432-s001.zip › antioxidants-2507142-supplementary.pdf]

Supplementary Material

Laricitrin 3-Rutinoside from Ginkgo biloba Fruits Protects Damage in TNF-α-Stimulated Normal Human Dermal Fibroblasts

Sullim Lee [1,†], Yea Jung Choi [2,†], Chen Huo [3,†], Akida Alishir [3], Ki Sung Kang [2], Il-Ho Park [4], Taesu Jang [5,*] and Ki Hyun Kim [3,*]

[1] Department of Life Science, College of Bio-Nano Technology, Gachon University, Seongnam 13120, Republic of Korea; sullimlee@gachon.ac.kr (S.L.)
[2] College of Korean Medicine, Gachon University, Seongnam 13120, Republic of Korea; domdada22@gachon.ac.kr (Y.J.C.); kkang@gachon.ac.kr (K.S.K.)
[3] School of Pharmacy, Sungkyunkwan University, Suwon 16419, Republic of Korea; huochen_0213@163.com (C.H.); akida.alishir@gmail.com (A.A.)
[4] College of Pharmacy, Sahmyook University, 815, Hwarang-ro, Nowon-gu, Seoul 01795, Republic of Korea; parkilho@syu.ac.kr (I.H.P.)
[5] Health Administration, Dankook University, Cheonan 31116, Republic of Korea; jangts@dankook.ac.kr (T.J.)
* Correspondence: jangts@dankook.ac.kr (T.J.); khkim83@skku.edu (K.H.K.); Tel.: +82-41-550-1476 (T.J.); +82-31-290-7700 (K.H.K.)
† These authors contributed equally to this work.

Figure S1. ^{1}H-NMR (CD_3OD, 850 MHz) spectrum of (*E*)-coniferin (**1**)........................ 4

Figure S2. UV chromatogram of LC/MS (A: monitored at 254 nm) and UV (B) and MS data (C: positive; D: negative) for **1**.. 5

Figure S3. ^{1}H-NMR (CD_3OD, 850 MHz) spectrum of syringin (**2**)................................ 6

Figure S4. UV chromatogram of LC/MS (A: monitored at 254 nm) and UV (B) and MS data (C: positive; D: negative) for **2**.. 7

Figure S5 ^{1}H-NMR (CD_3OD, 850 MHz) spectrum of 4-hydroxybenzoic acid 4-*O*-β-D-glucopyranoside (**3**).. 8

Figure S6. UV chromatogram of LC/MS (A: monitored at 254 nm) and UV (B) and MS data (C: positive; D: negative) for **3**.. 9

Figure S7. ^{1}H-NMR (CD_3OD, 850 MHz) spectrum of vanillic acid 4-*O*-β-D-glucopyranoside (**4**).. 10

Figure S8. UV chromatogram of LC/MS (A: monitored at 315 nm) and UV (B) and MS data (C: positive; D: negative) for **4**.. 11

Figure S9. ^{1}H-NMR (CD_3OD, 850 MHz) spectrum of glucosyringic acid (**5**). 12

Figure S10. UV chromatogram of LC/MS (A: monitored at 315 nm) and UV (B) and MS data (C: positive; D: negative) for **5**.. 13

Figure S11. ^{1}H-NMR (CD_3OD, 850 MHz) spectrum of (*E*)-ferulic acid 4-*O*-β-D-glucoside (**6**).. 14

Figure S12. UV chromatogram of LC/MS (A: monitored at 315 nm) and UV (B) and MS data (C: positive; D: negative) for **6** 15

Figure S13. ^{1}H-NMR (CD_3OD, 850 MHz) spectrum of (*E*)-sinapic acid 4-*O*-β-D-glucopyranoside (**7**).. 16

Figure S14. UV chromatogram of LC/MS (A: monitored at 315 nm) and UV (B) and MS data (C: positive; D: negative) for **7**.. 17

Figure S15. ^{1}H-NMR (CD_3OD, 850 MHz) spectrum of ginkgotoxin-5-glucoside (**8**). 18

Figure S16. UV chromatogram of LC/MS (A: monitored at 315 nm) and UV (B) and MS data (C: positive; D: negative) for **8**.. 19

Figure S17. ^{1}H-NMR (CD_3OD, 850 MHz) spectrum of ginkgopanoside (**9**). 20

Figure S18. UV chromatogram of LC/MS (A: monitored at 254 nm) and UV (B) and MS data (C: positive; D: negative) for **9**.. 21

Figure S19. ^{1}H-NMR (CD_3OD, 850 MHz) spectrum of (*Z*)-4-coumaric acid 4-*O*-β-D-glucopyranoside (**10**). 22

Figure S20. UV chromatogram of LC/MS (A: monitored at 254 nm) and UV (B) and MS data (C: positive; D: negative) for **10**. 23

Figure S21. ^{1}H-NMR (CD_3OD, 850 MHz) spectrum of (1'*R*,2'*S*,5'*R*,8'*S*,2'*Z*,4'*E*)-dihydrophaseic acid 3'-*O*-β-D-glucopyranoside (**11**). 24

Figure S22. UV chromatogram of LC/MS (A: monitored at 254 nm) and UV (B) and MS data (C: positive; D: negative) for **11**. 25

Figure S23. ^{1}H-NMR (CD_3OD, 850 MHz) spectrum of eucomic acid (**12**). 26

Figure S24. UV chromatogram of LC/MS (A: monitored at 254 nm) and UV (B) and MS data (C: positive; D: negative) for **12**. 27

Figure S25. ^{1}H-NMR (CD_3OD, 850 MHz) spectrum of rutin (**13**). 28

Figure S26. UV chromatogram of LC/MS (A: monitored at 254 nm) and UV (B) and MS data (C: positive; D: negative) for **13**. 29

Figure S27. ^{1}H-NMR (CD_3OD, 850 MHz) spectrum of laricitrin 3-rutinoside (**14**). ... 30

Figure S28. UV chromatogram of LC/MS (A: monitored at 254 nm) and UV (B) and MS data (C: positive; D: negative) for **14**. 31

Figure S29. Effect of the extract on NHDF viability 32

Figure S30. Effects of the extract on TNF-α-induced intercellular reactive oxygen species (ROS) generation 33

Figure S31. Effect of the extract on MMP-1 and COLIA1 protein expression in tumor necrosis factor-α (TNF-α)-stimulated normal human dermal fibroblasts (NHDFs) 34

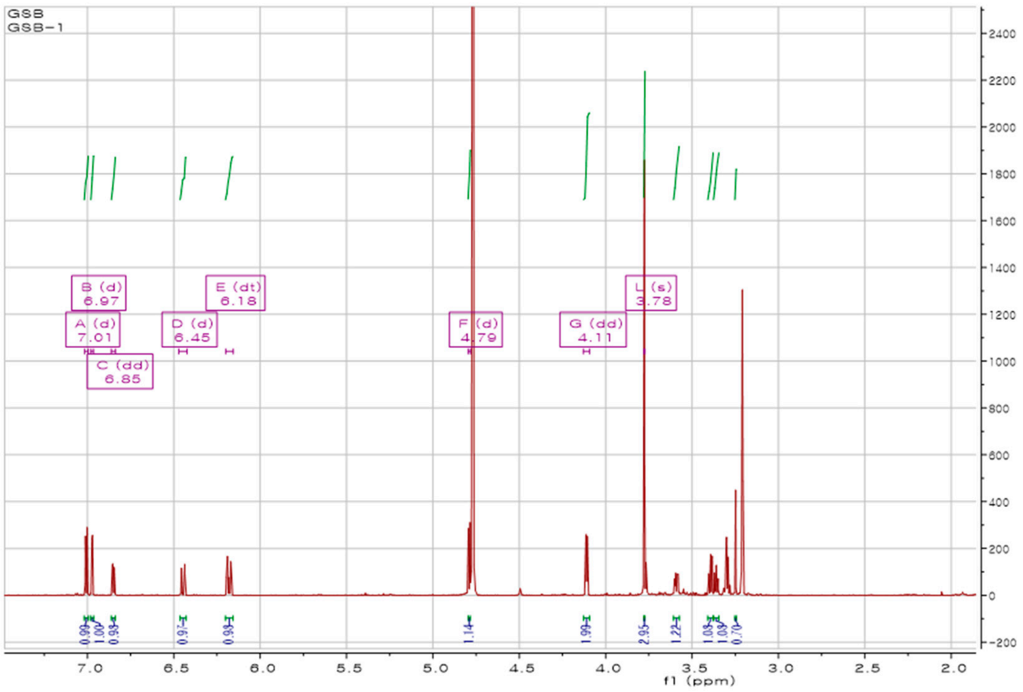

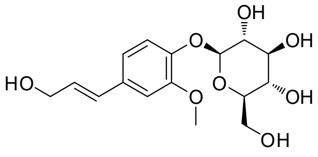

1

Figure S1. ^{1}H-NMR (CD_3OD, 850 MHz) spectrum of (*E*)-coniferin (**1**).

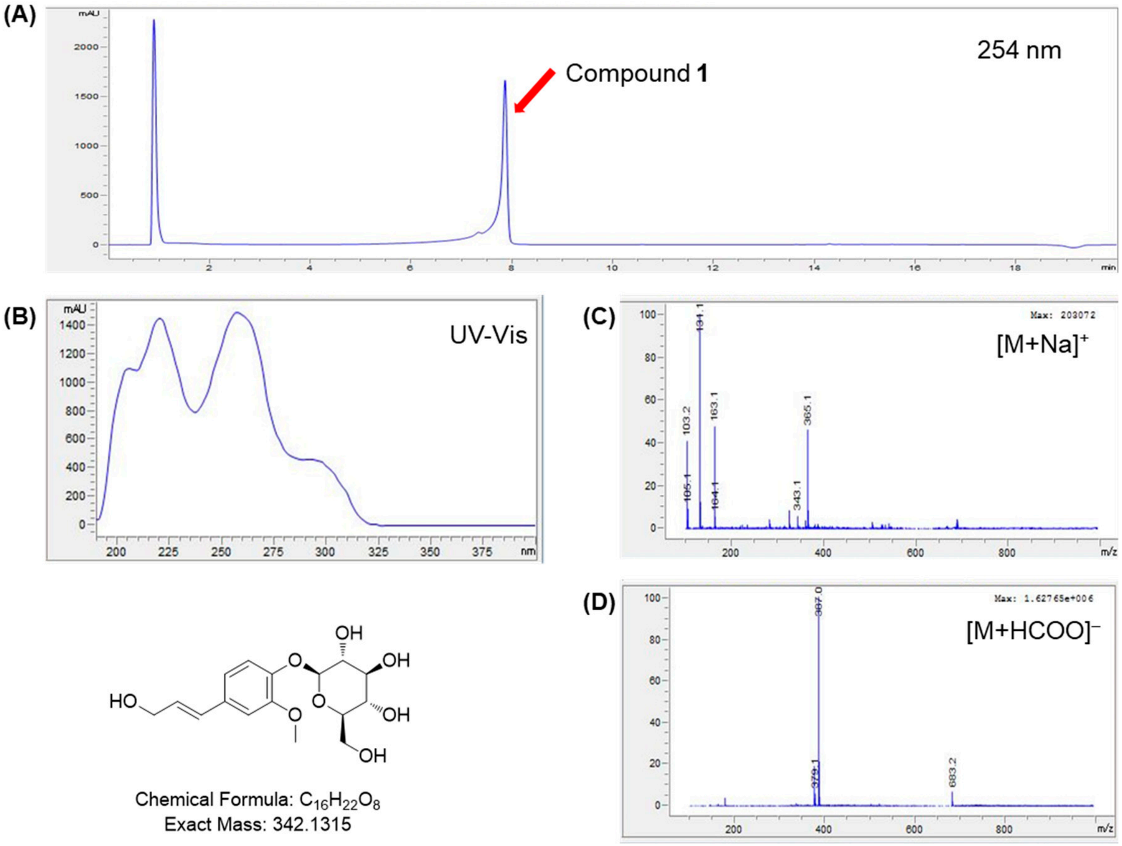

Figure S2. UV chromatogram of LC/MS (A: monitored at 254 nm) and UV (B) and MS data (C: positive; D: negative) for **1**.

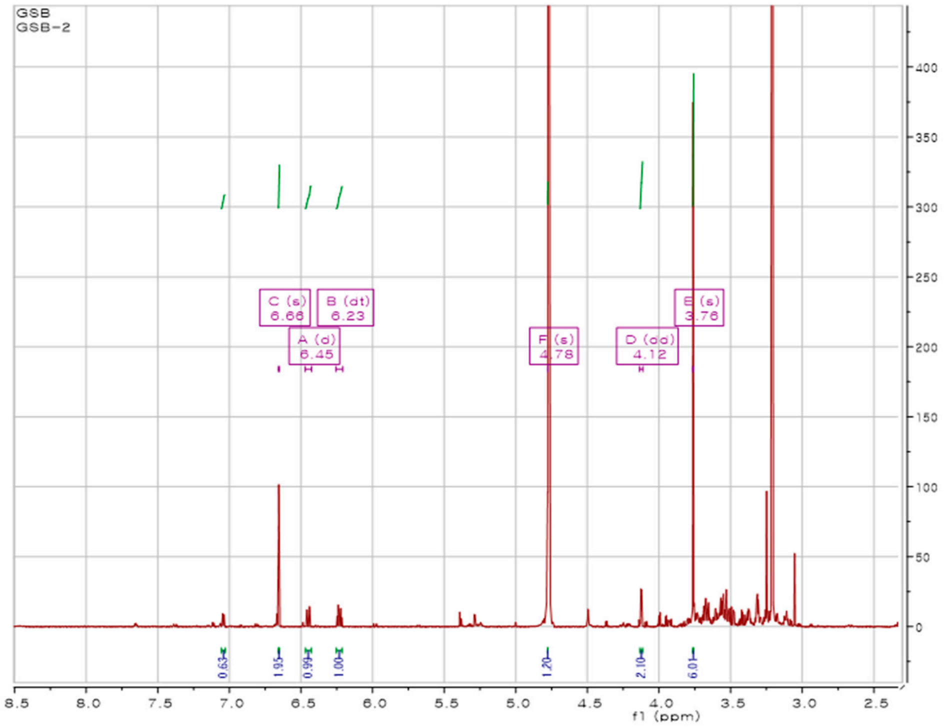

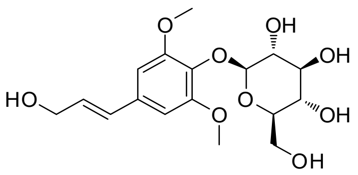

2

Figure S3. ^{1}H-NMR (CD_3OD, 850 MHz) spectrum of syringin (**2**).

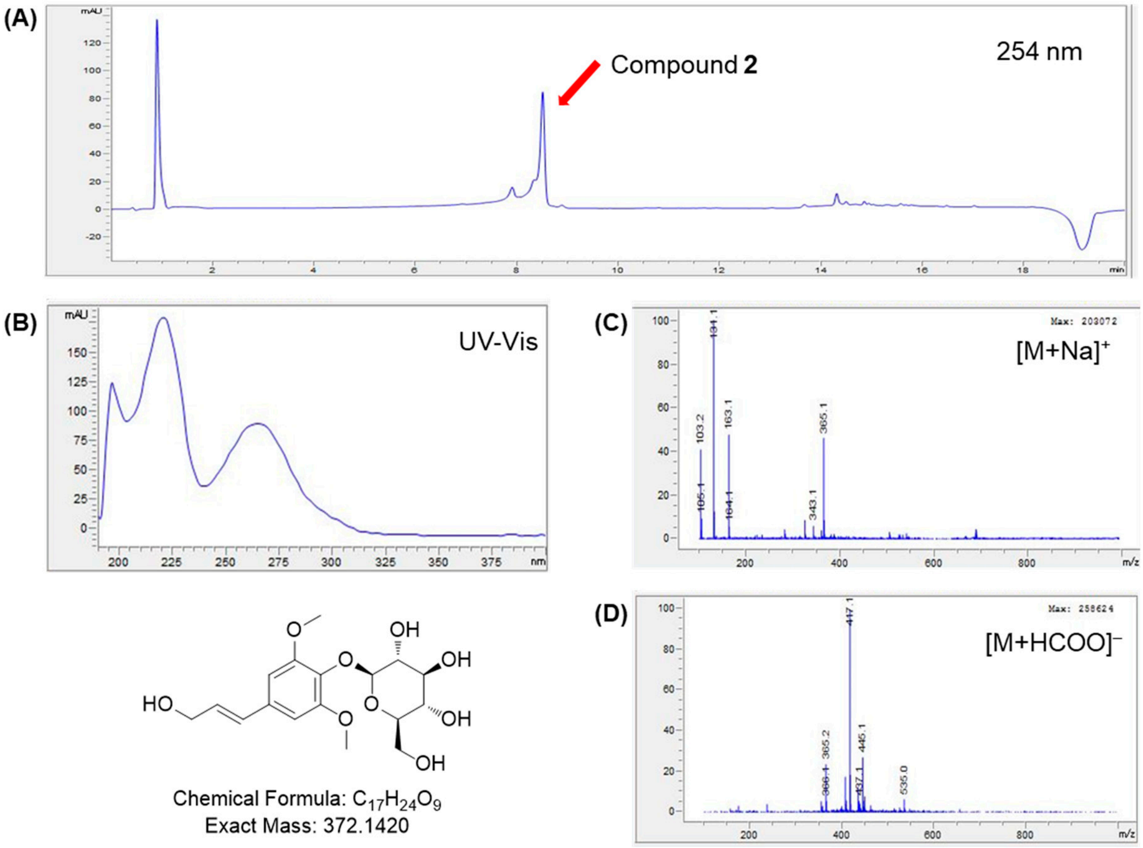

Figure S4. UV chromatogram of LC/MS (A: monitored at 254 nm) and UV (B) and MS data (C: positive; D: negative) for **2**.

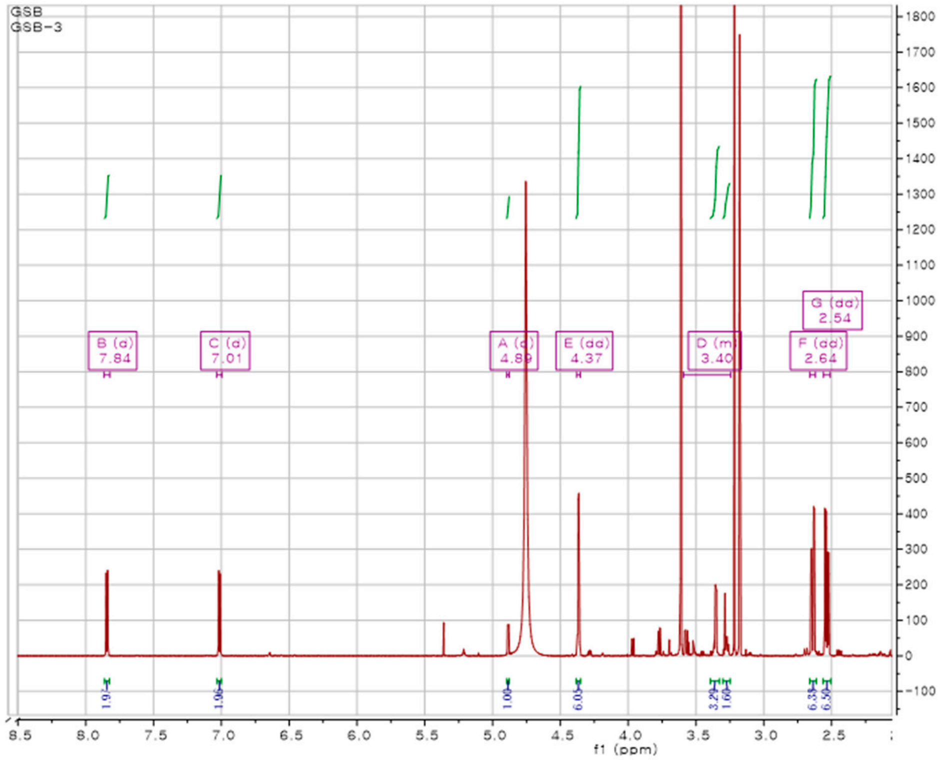

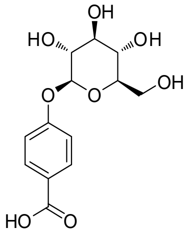

3

Figure S5 ^{1}H-NMR (CD_3OD, 850 MHz) spectrum of 4-hydroxybenzoic acid 4-*O*-β-D-glucopyranoside (**3**).

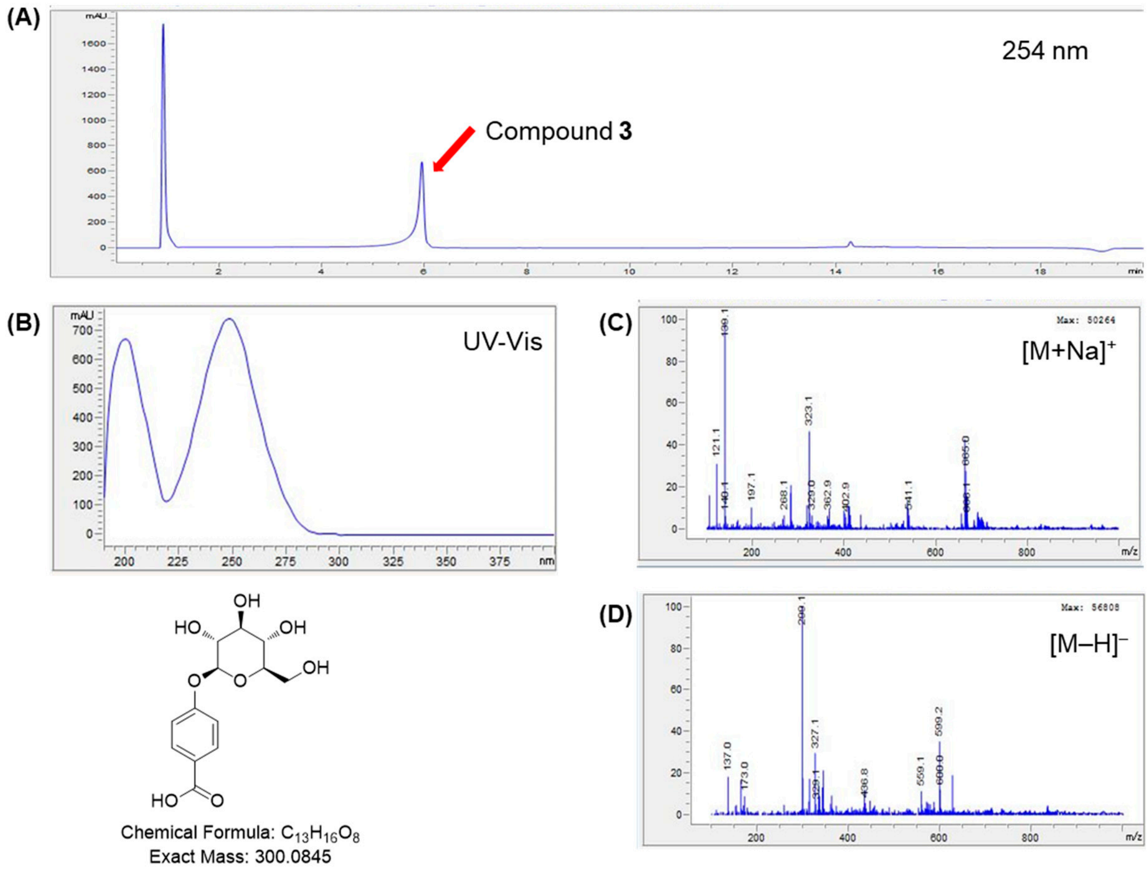

Figure S6. UV chromatogram of LC/MS (A: monitored at 254 nm) and UV (B) and MS data (C: positive; D: negative) for **3**.

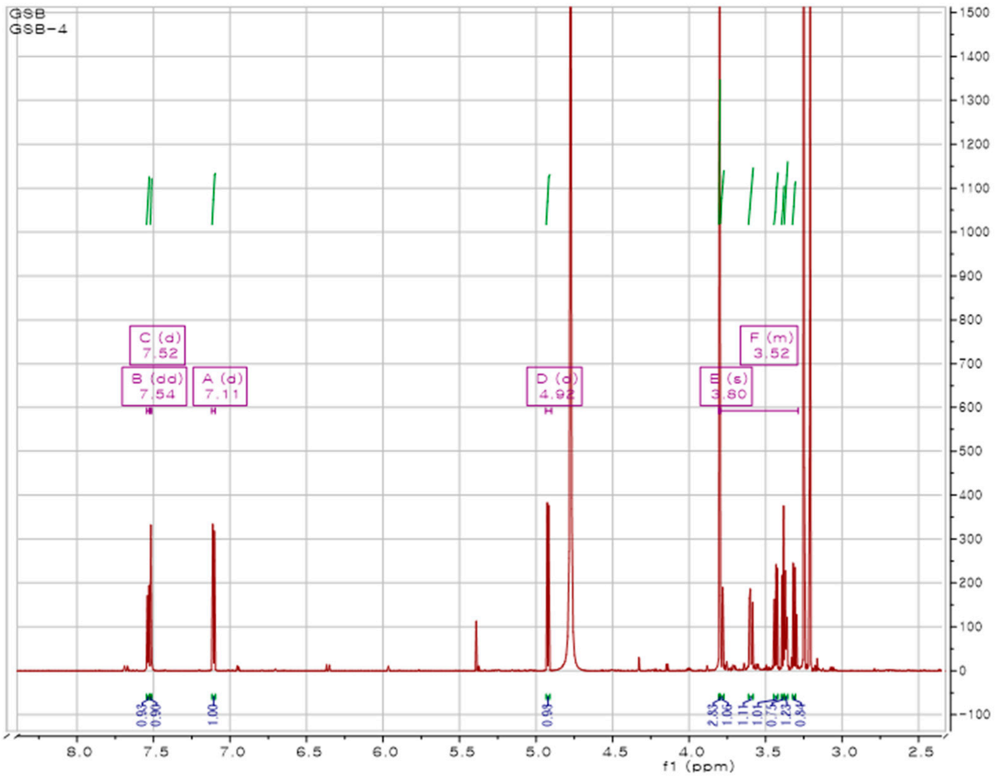

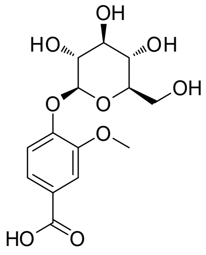

4

Figure S7. ^{1}H-NMR (CD_3OD, 850 MHz) spectrum of vanillic acid 4-*O*-β-D-glucopyranoside (**4**).

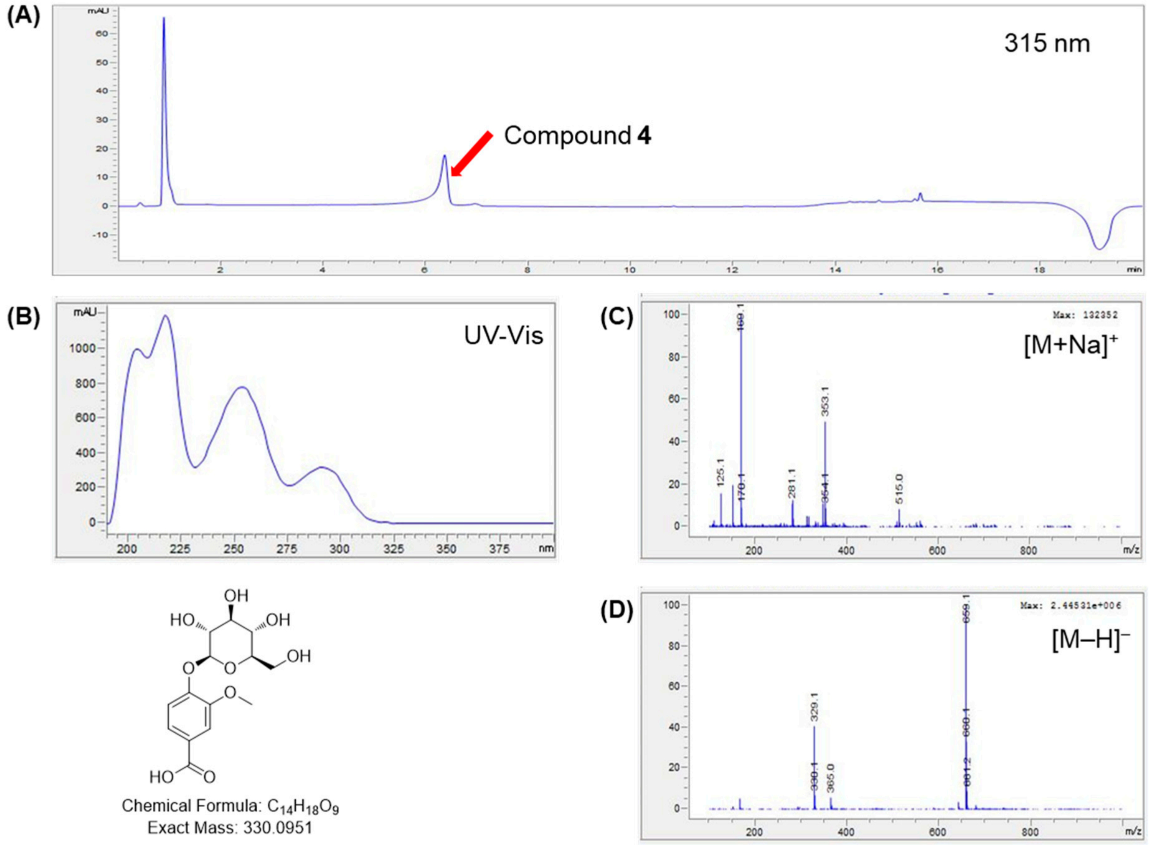

Figure S8. UV chromatogram of LC/MS (A: monitored at 315 nm) and UV (B) and MS data (C: positive; D: negative) for **4**.

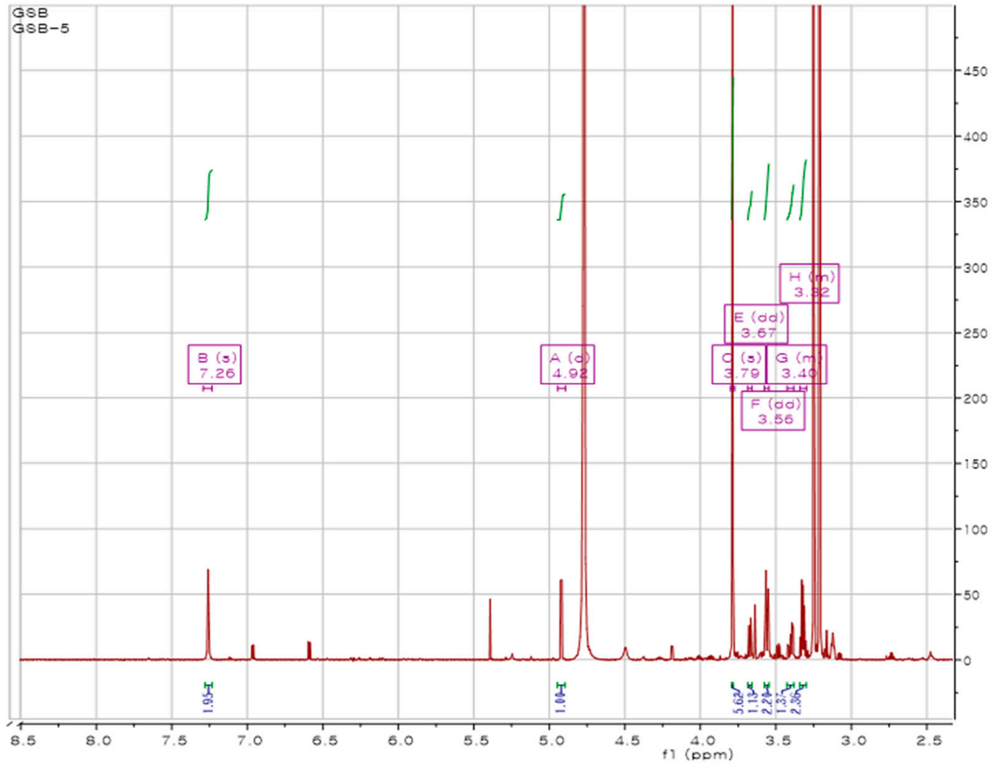

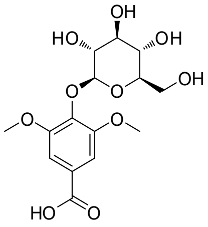

5

Figure S9. ^1H-NMR (CD_3OD, 850 MHz) spectrum of glucosyringic acid (**5**).

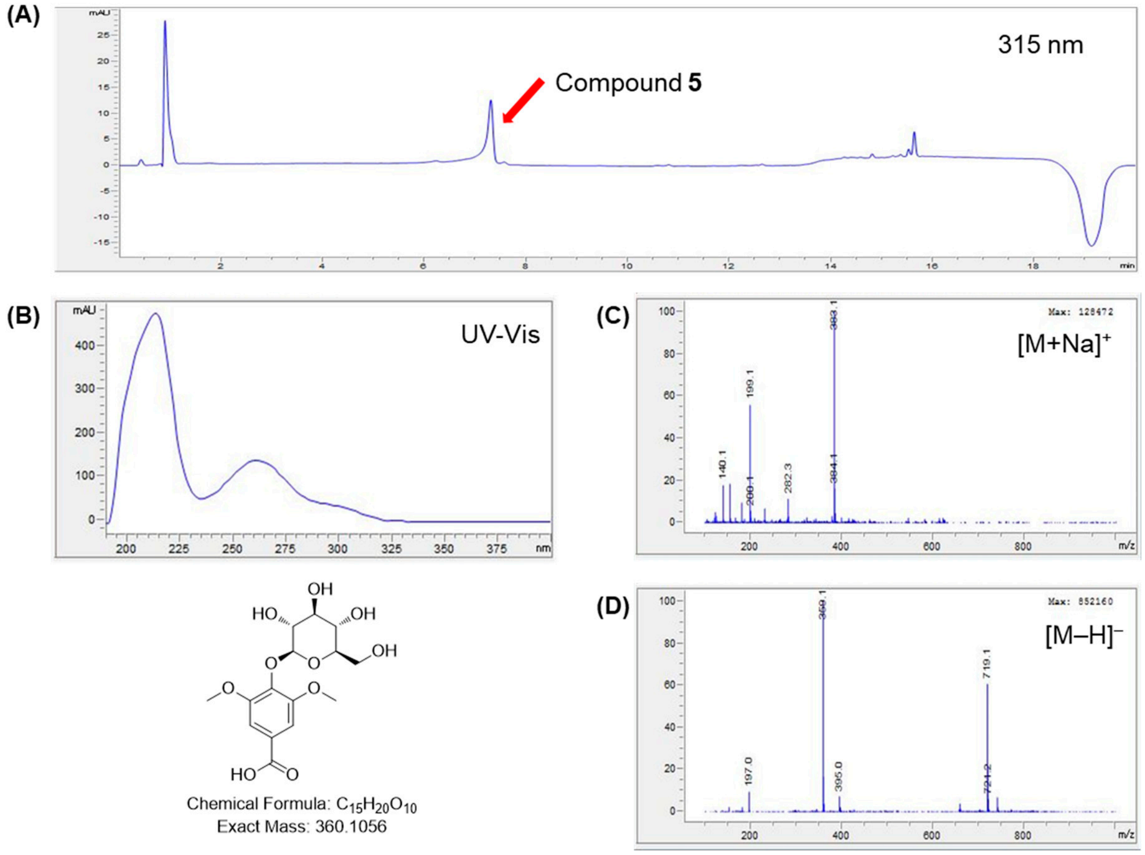

Figure S10. UV chromatogram of LC/MS (A: monitored at 315 nm) and UV (B) and MS data (C: positive; D: negative) for **5**.

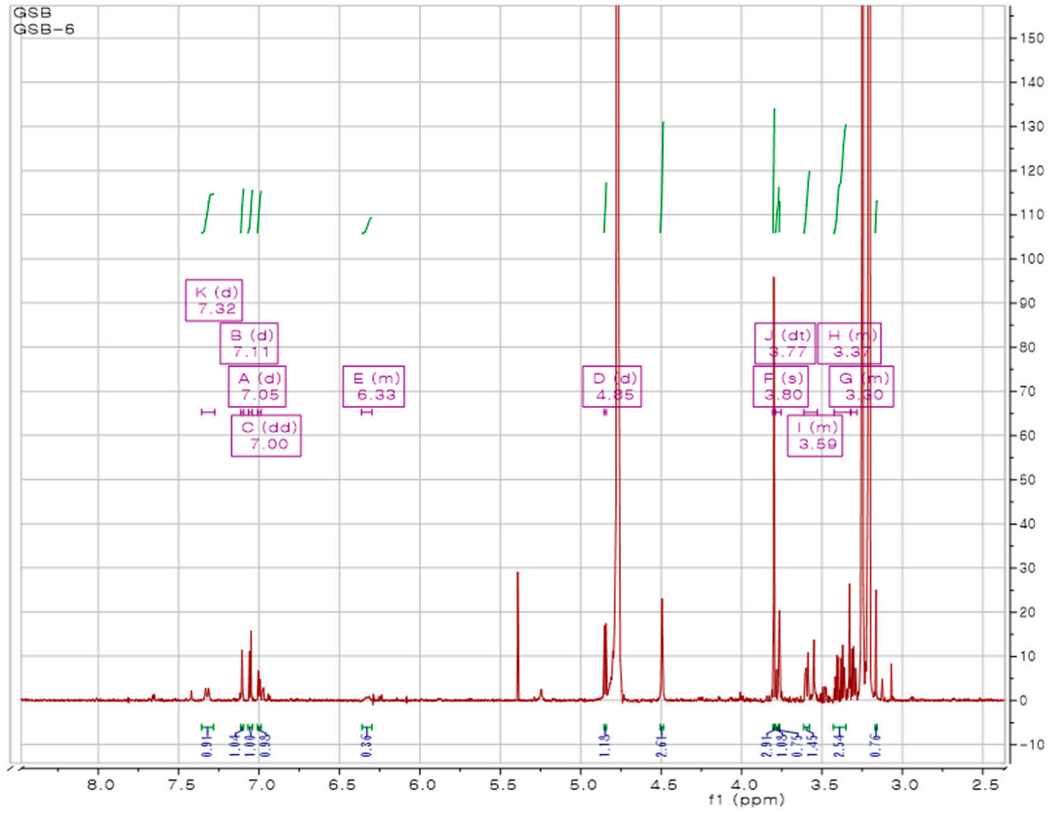

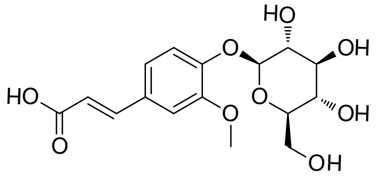

6

Figure S11. ^{1}H-NMR (CD_3OD, 850 MHz) spectrum of (*E*)-ferulic acid 4-*O*-β-D-glucoside (**6**).

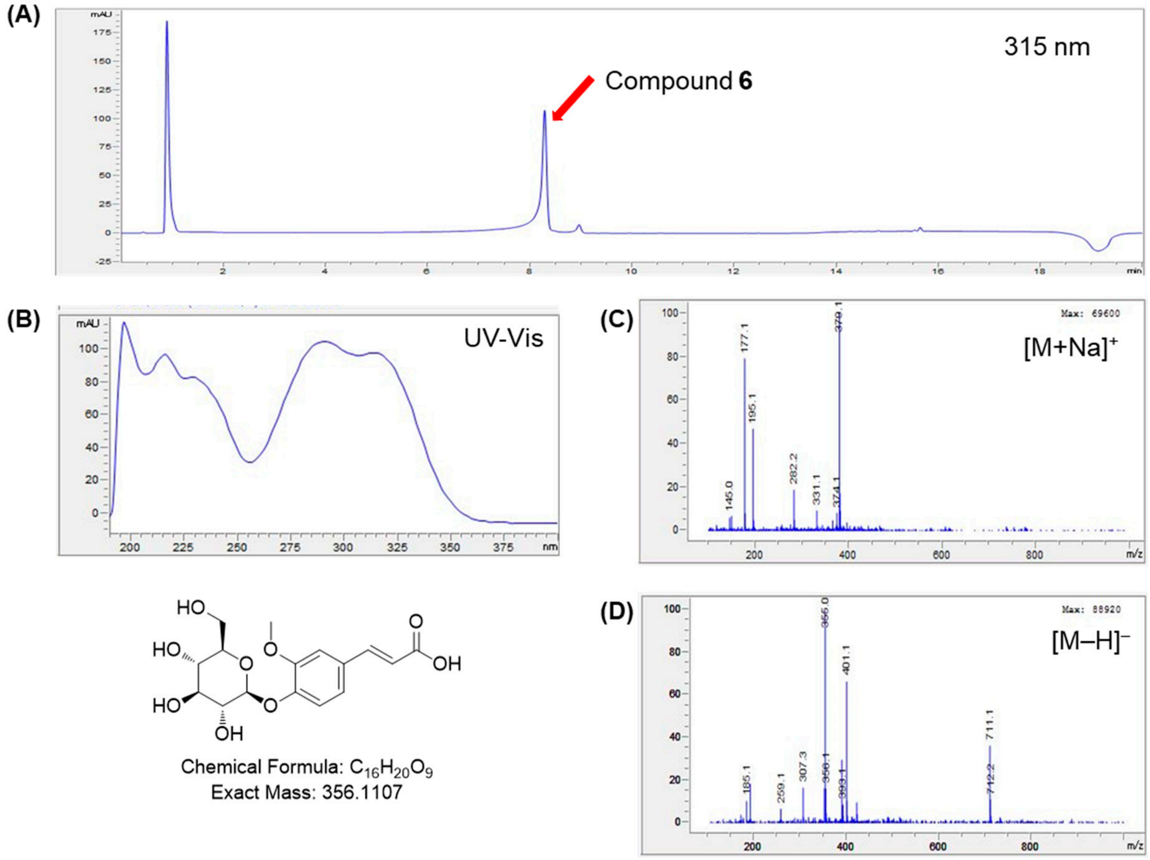

Figure S12. UV chromatogram of LC/MS (A: monitored at 315 nm) and UV (B) and MS data (C: positive; D: negative) for **6**.

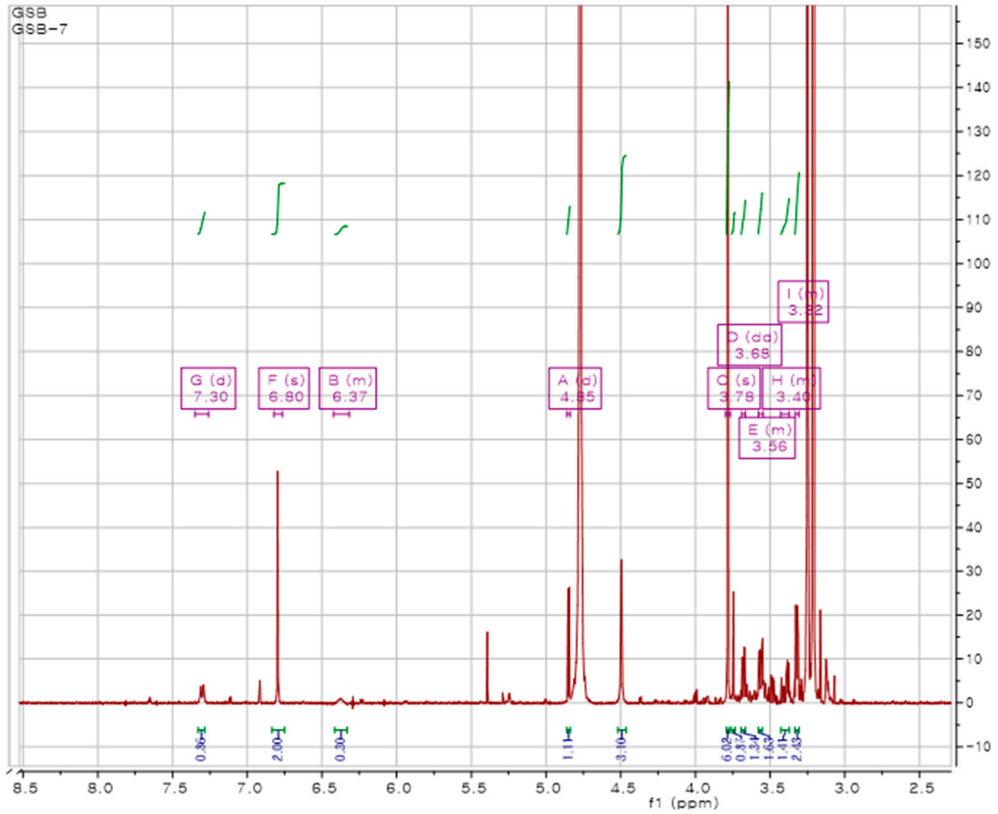

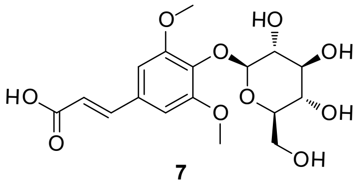

7

Figure S13. ¹H-NMR (CD_3OD, 850 MHz) spectrum of (*E*)-sinapic acid 4-*O*-β-D-glucopyranoside (**7**).

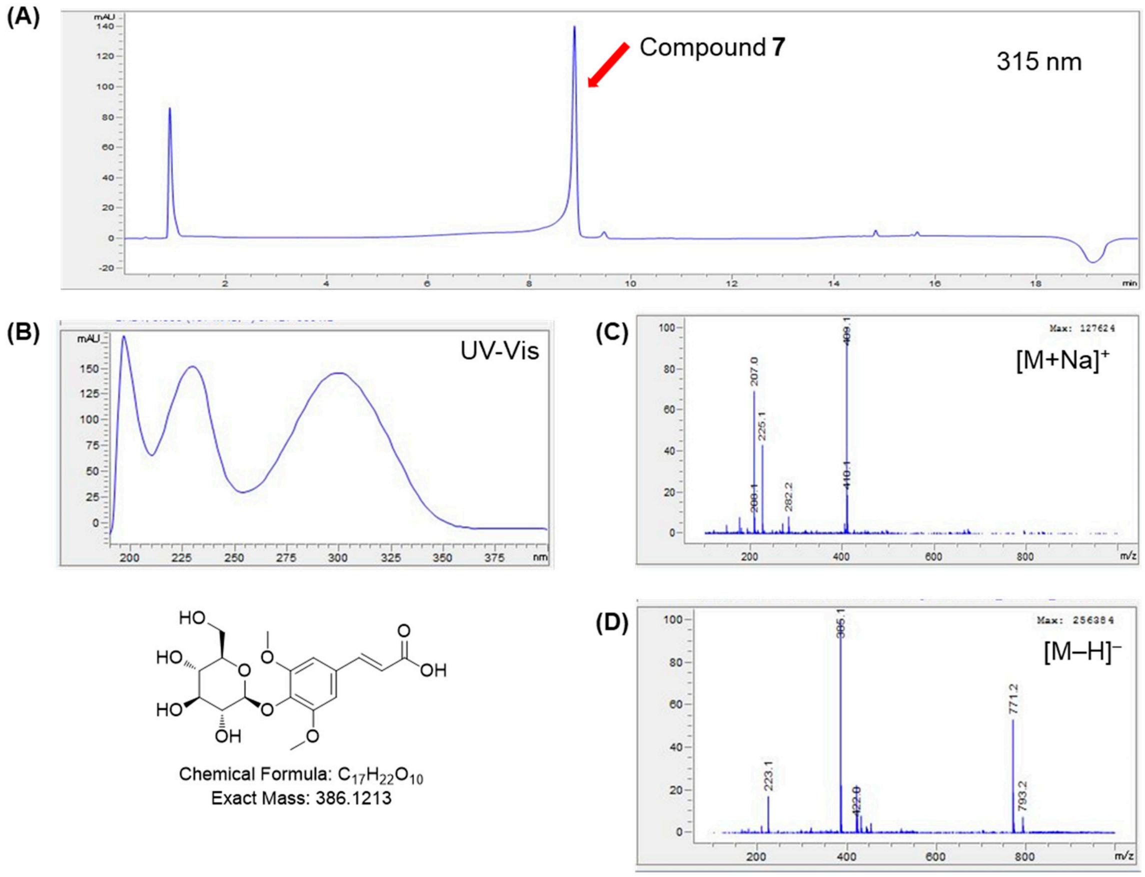

Figure S14. UV chromatogram of LC/MS (A: monitored at 315 nm) and UV (B) and MS data (C: positive; D: negative) for **7**.

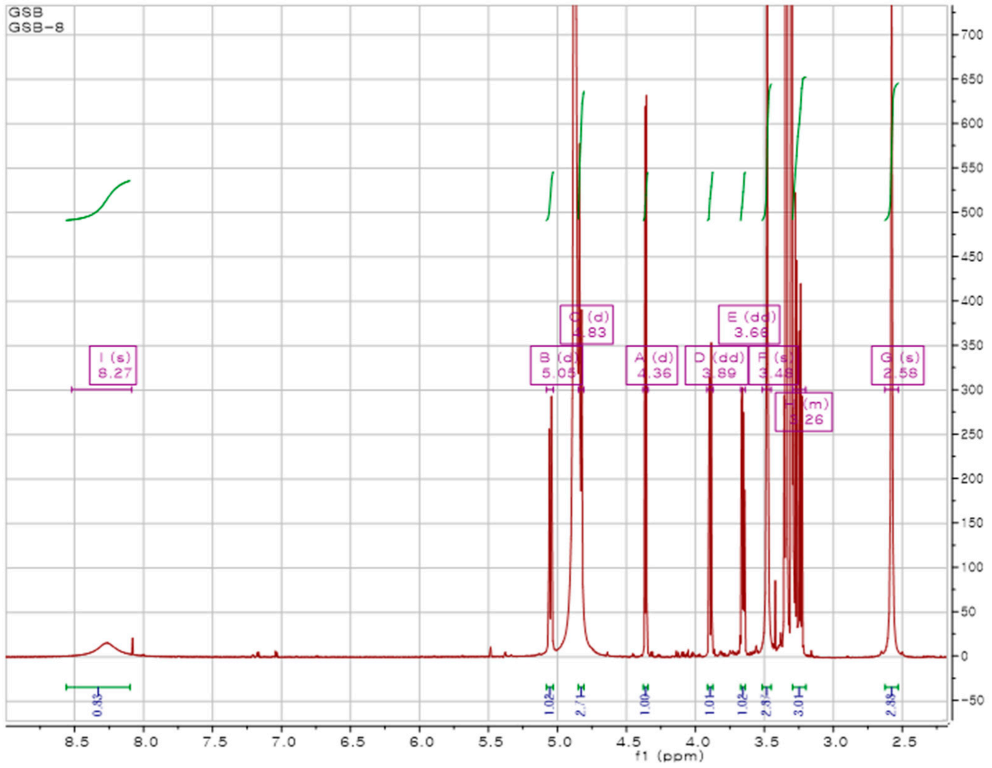

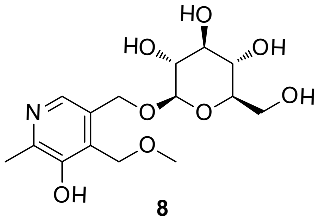

8

Figure S15. ^{1}H-NMR (CD_3OD, 850 MHz) spectrum of ginkgotoxin-5-glucoside (**8**).

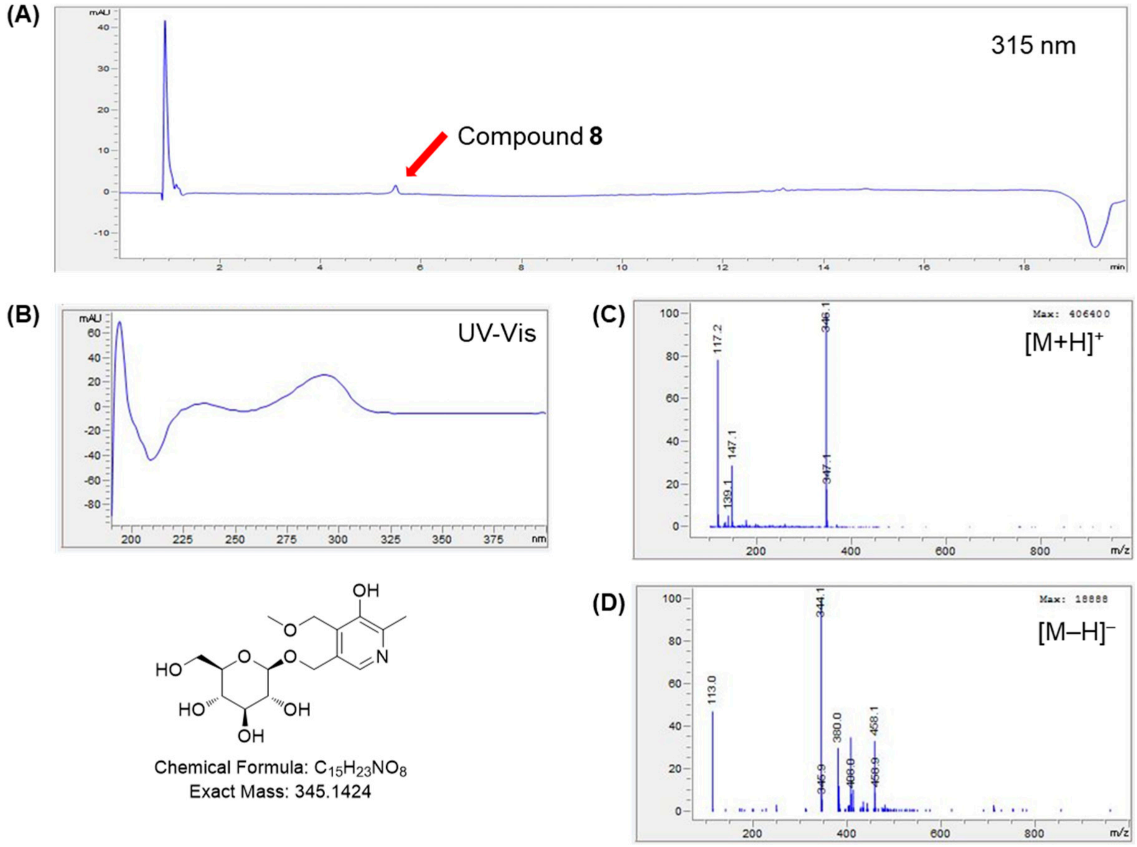

Figure S16. UV chromatogram of LC/MS (A: monitored at 315 nm) and UV (B) and MS data (C: positive; D: negative) for **8**.

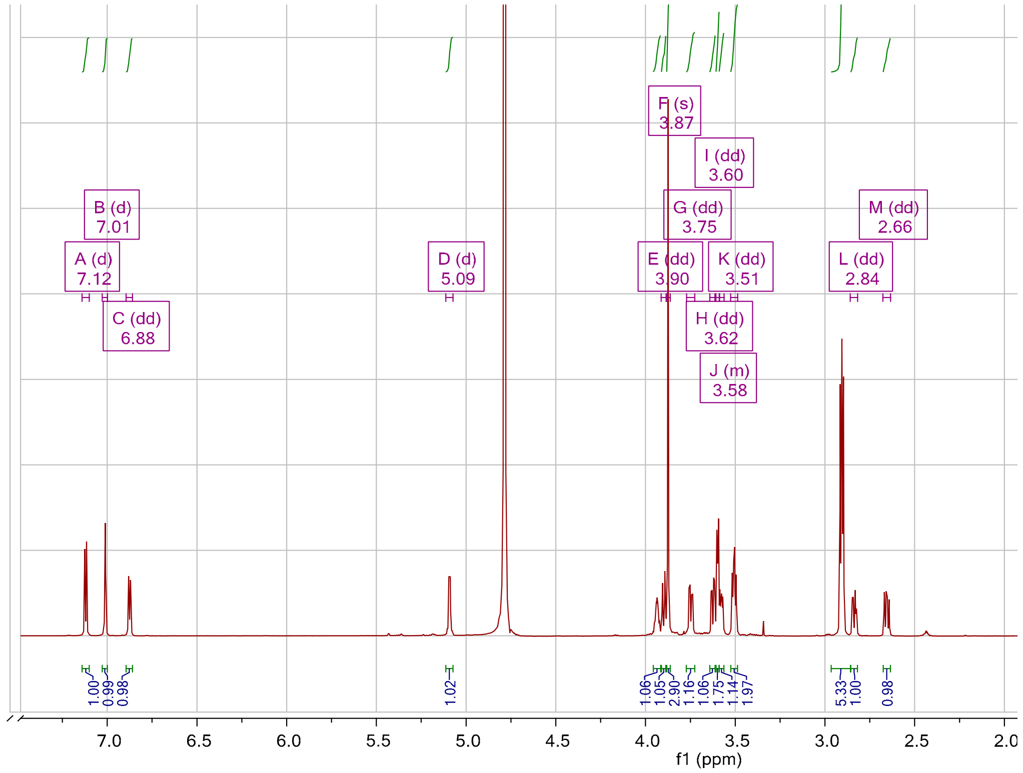

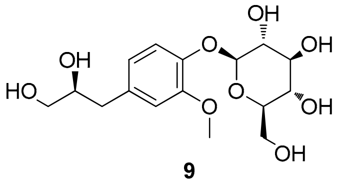

9

Figure S17. ^{1}H-NMR (CD_3OD, 850 MHz) spectrum of ginkgopanoside (**9**).

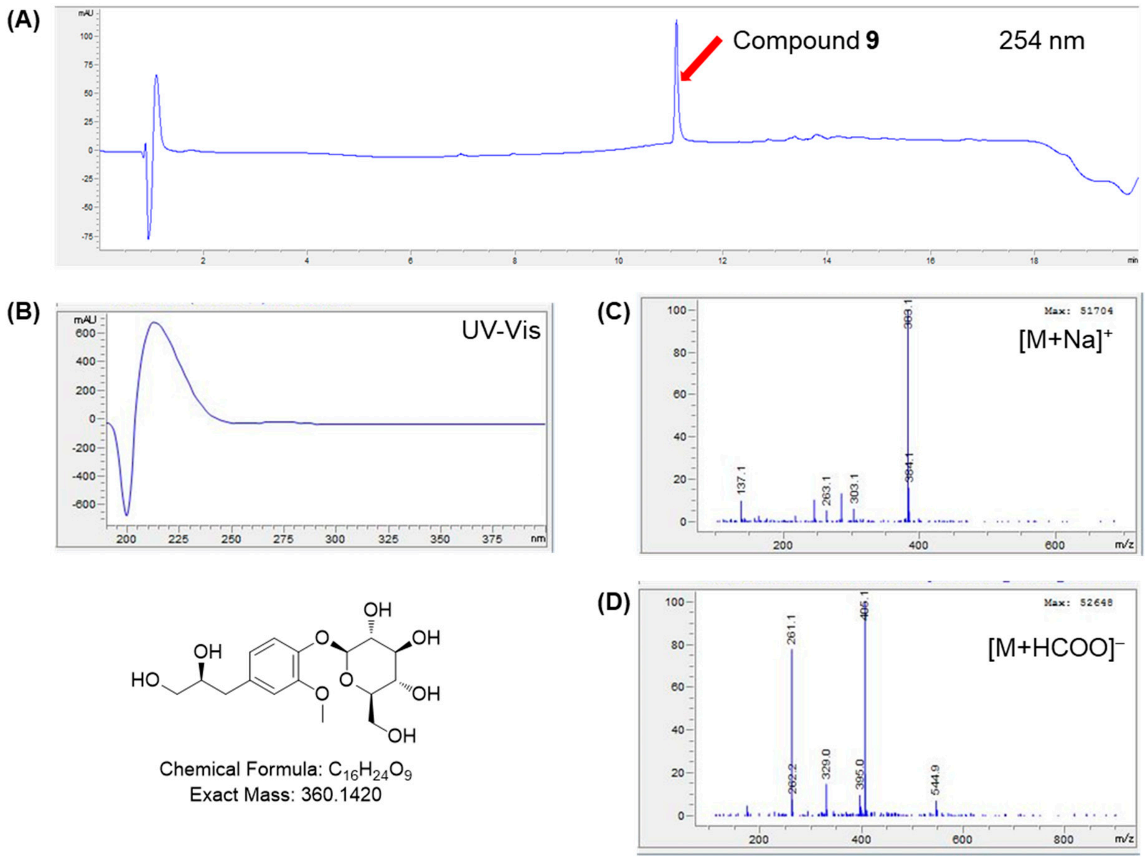

Figure S18. UV chromatogram of LC/MS (A: monitored at 254 nm) and UV (B) and MS data (C: positive; D: negative) for **9**.

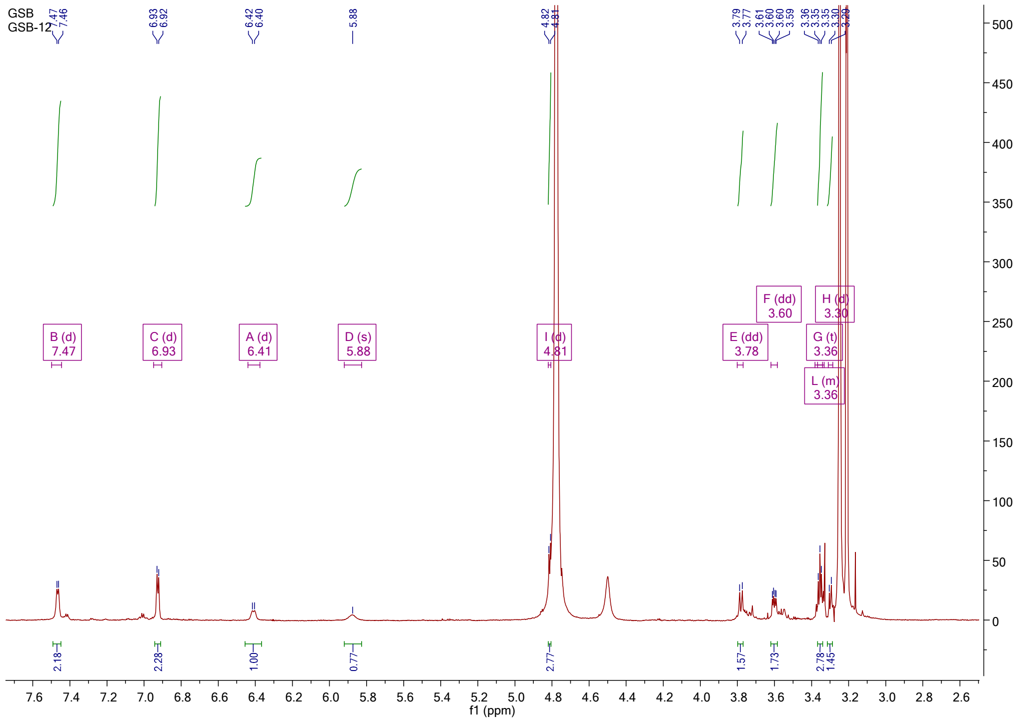

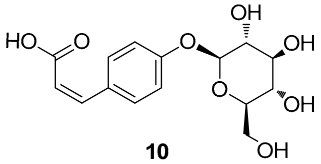

10

Figure S19. ^{1}H-NMR (CD_3OD, 850 MHz) spectrum of (*Z*)-4-coumaric acid 4-*O*-β-D-glucopyranoside (**10**).

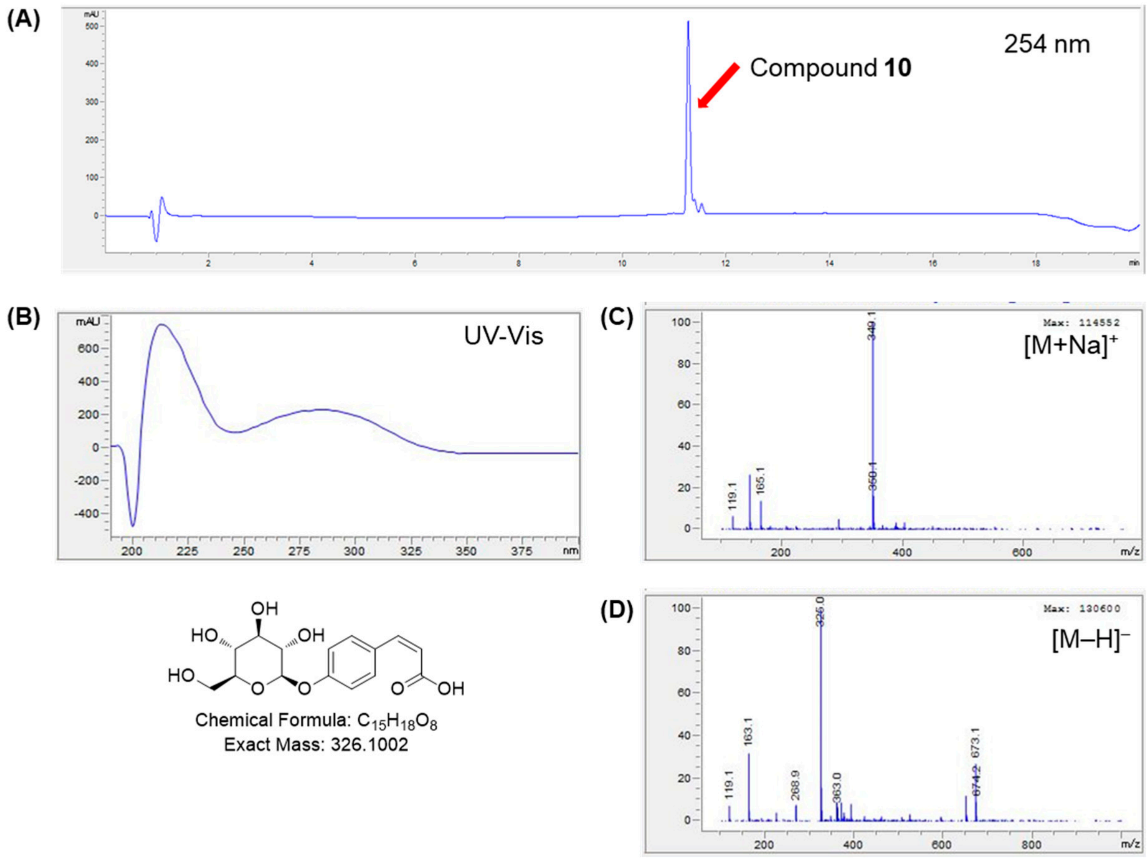

Figure S20. UV chromatogram of LC/MS (A: monitored at 254 nm) and UV (B) and MS data (C: positive; D: negative) for **10**.

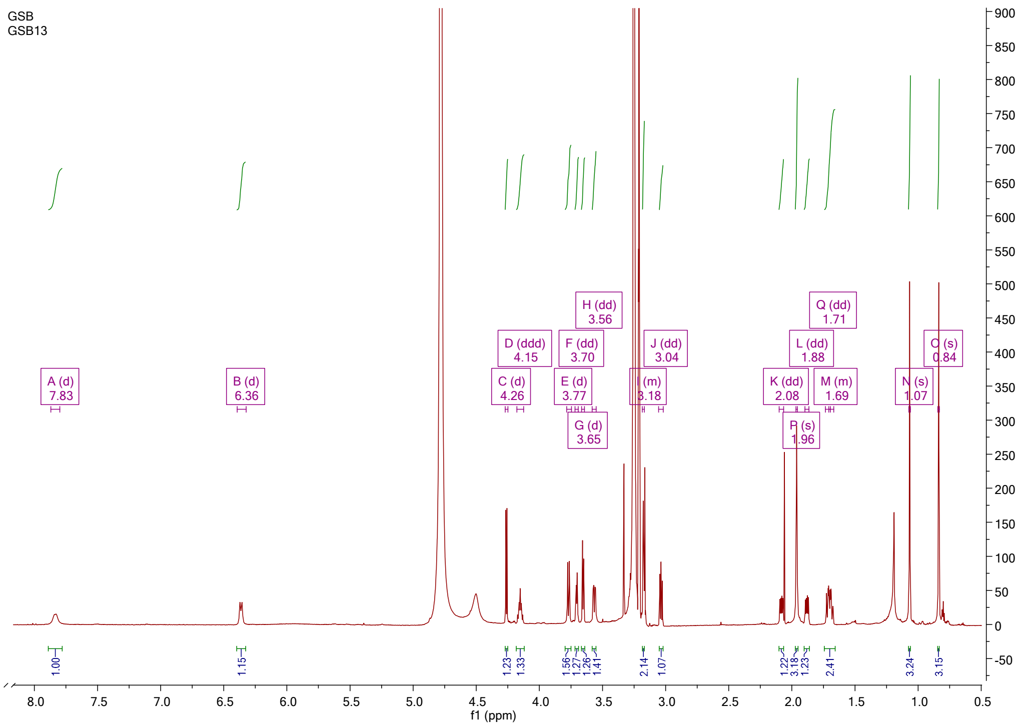

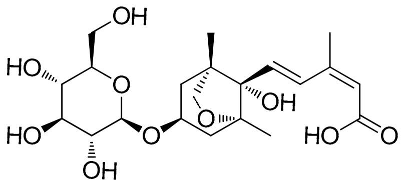

11

Figure S21. ^{1}H-NMR (CD₃OD, 850 MHz) spectrum of (1′*R*,2′*S*,5′*R*,8′*S*,2′*Z*,4′*E*)-dihydrophaseic acid 3'-*O*-β-D-glucopyranoside (**11**).

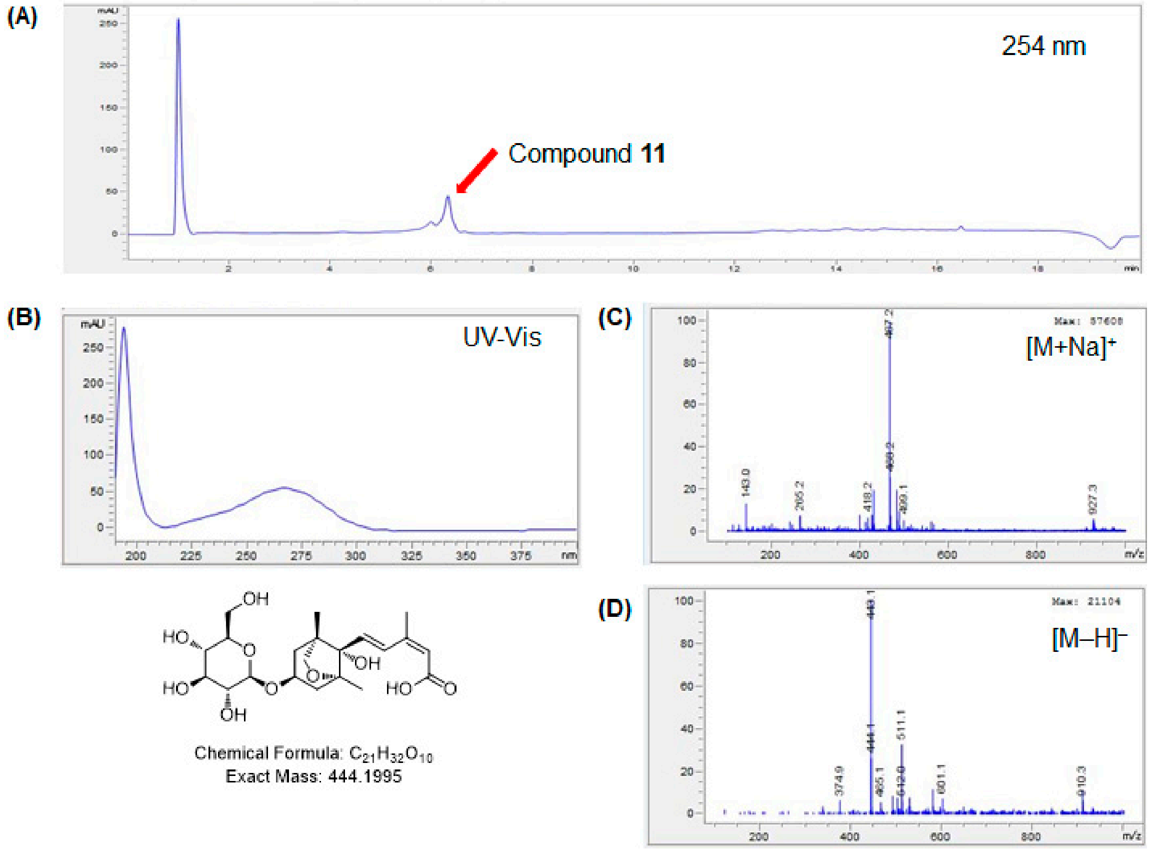

Figure S22. UV chromatogram of LC/MS (A: monitored at 254 nm) and UV (B) and MS data (C: positive; D: negative) for **11**.

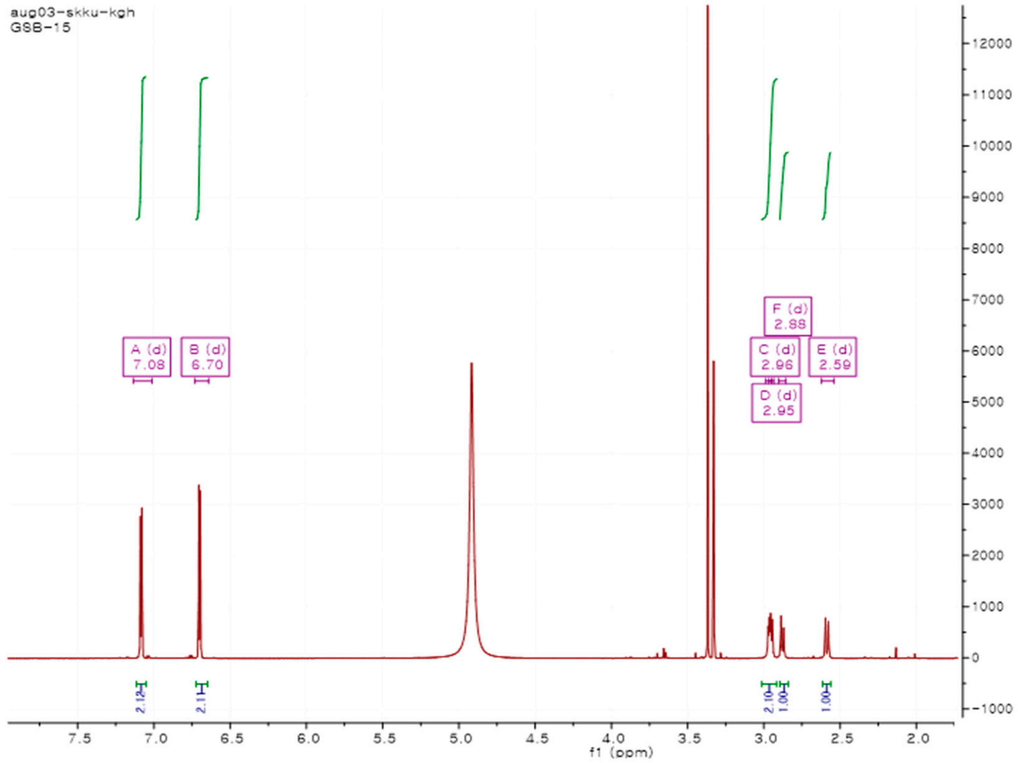

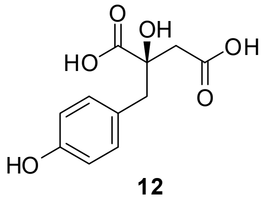

12

Figure S23. ^{1}H-NMR (CD_3OD, 850 MHz) spectrum of eucomic acid (**12**).

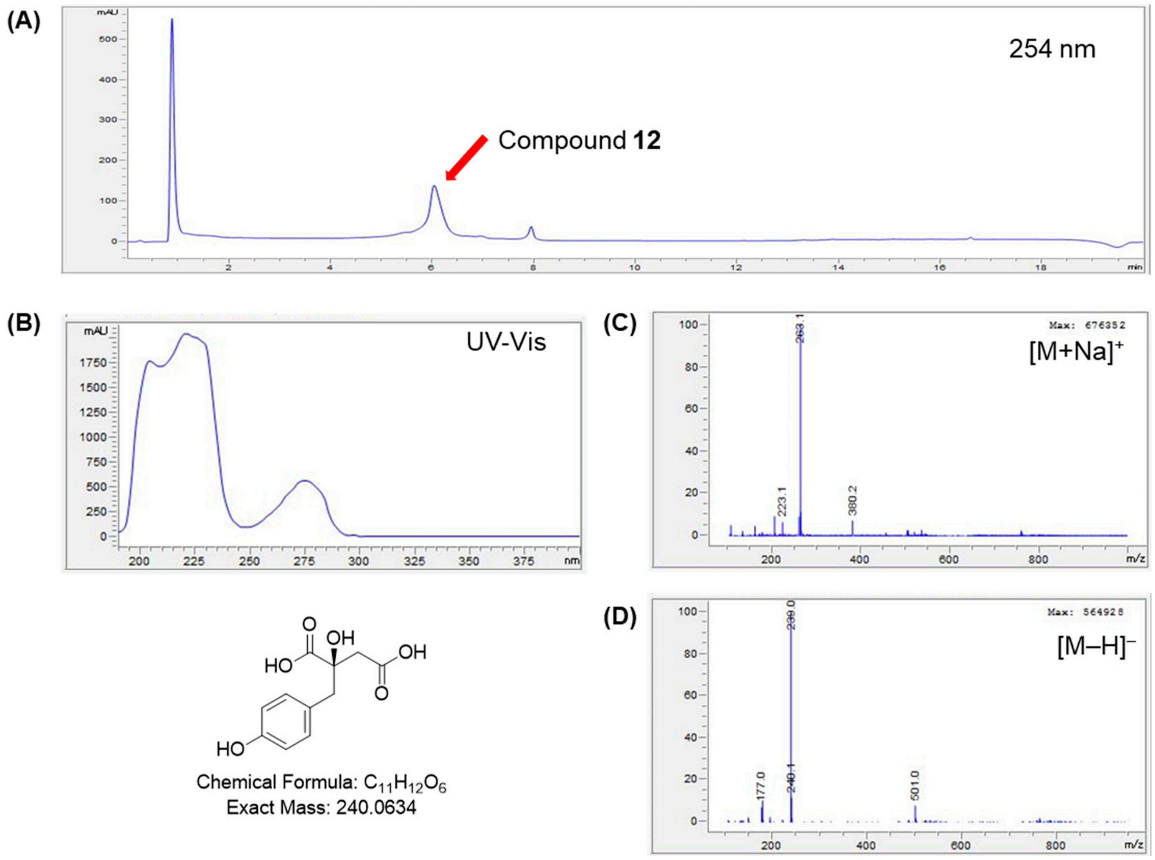

Figure S24. UV chromatogram of LC/MS (A: monitored at 254 nm) and UV (B) and MS data (C: positive; D: negative) for **12**.

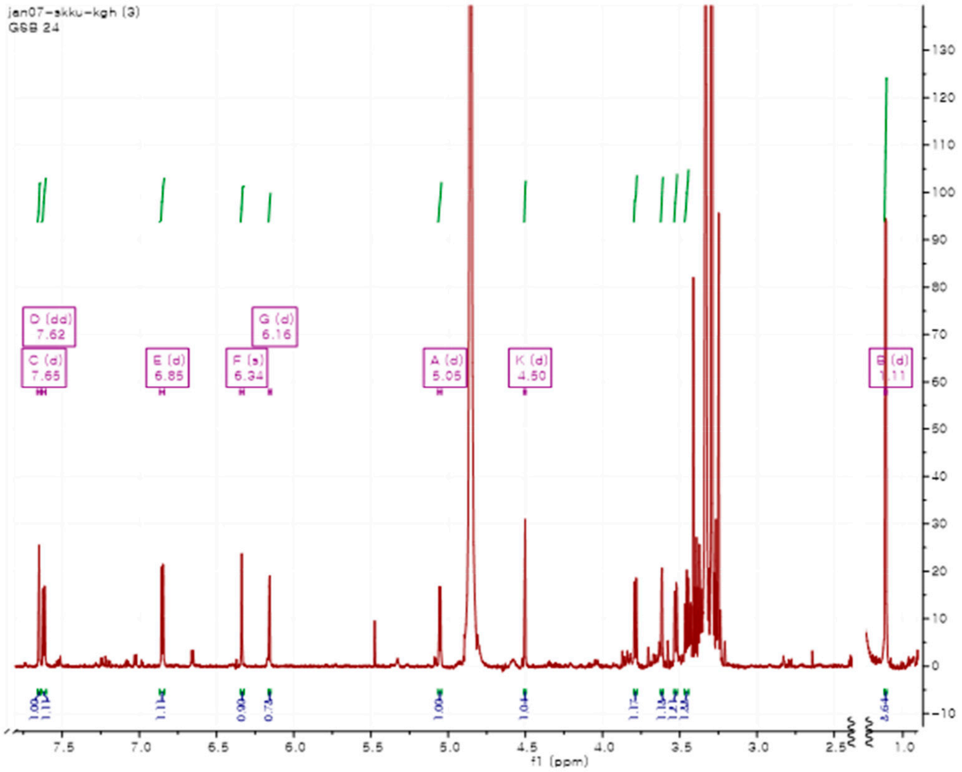

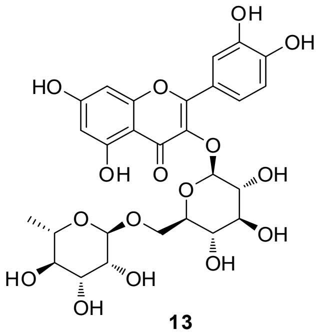

13

Figure S25. ^{1}H-NMR (CD_3OD, 850 MHz) spectrum of rutin (**13**).

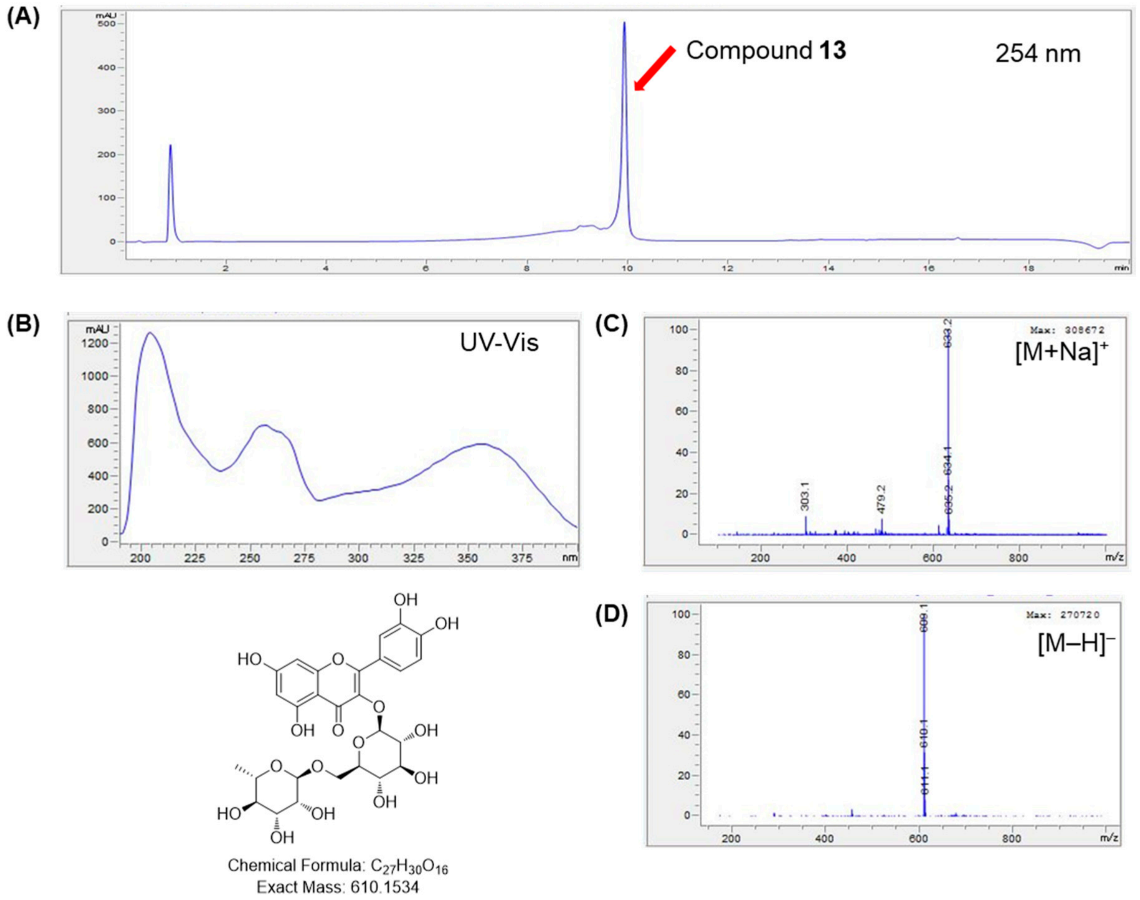

Figure S26. UV chromatogram of LC/MS (A: monitored at 254 nm) and UV (B) and MS data (C: positive; D: negative) for **13**.

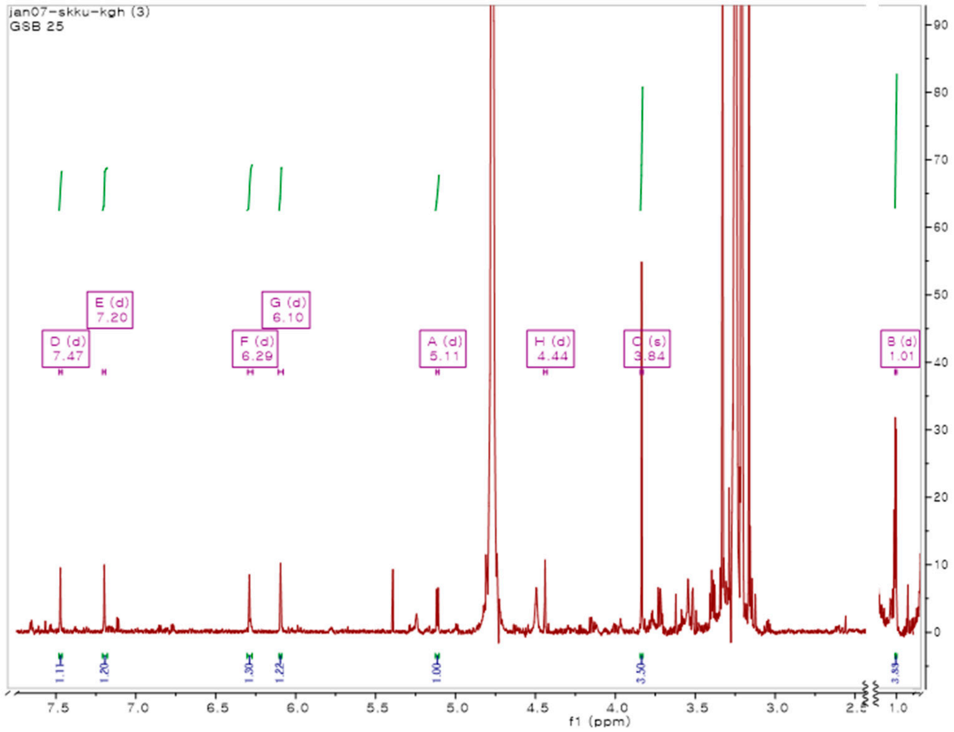

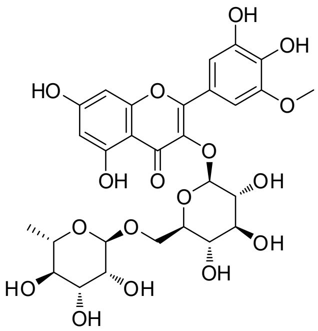

14

Figure S27. ^{1}H-NMR (CD_3OD, 850 MHz) spectrum of laricitrin 3-rutinoside (**14**).

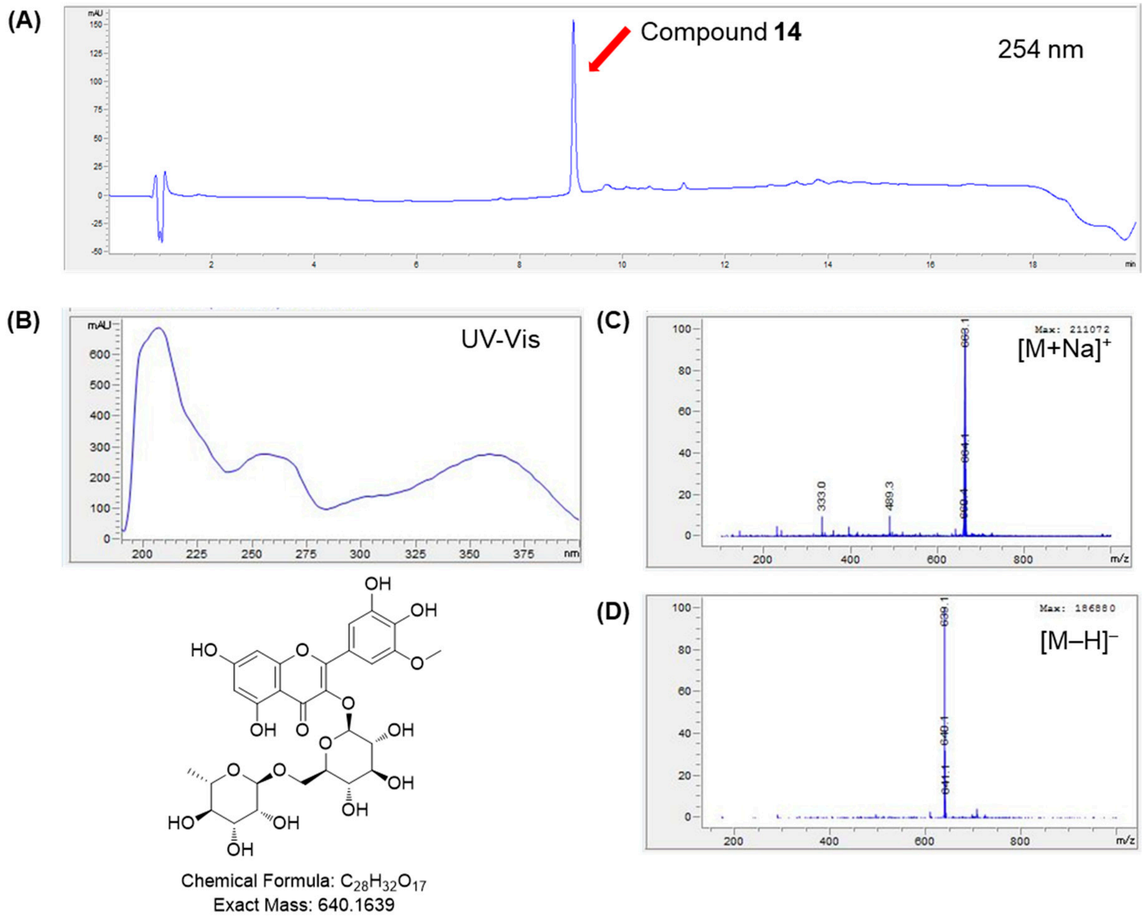

Figure S28. UV chromatogram of LC/MS (A: monitored at 254 nm) and UV (B) and MS data (C: positive; D: negative) for **14**.

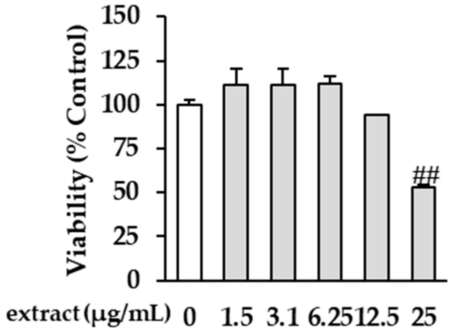

Figure S29. Effect of the extract on NHDF viability. Data are presented as mean ± SEM (n = 2). ## $p < 0.01$ compared with the control group.

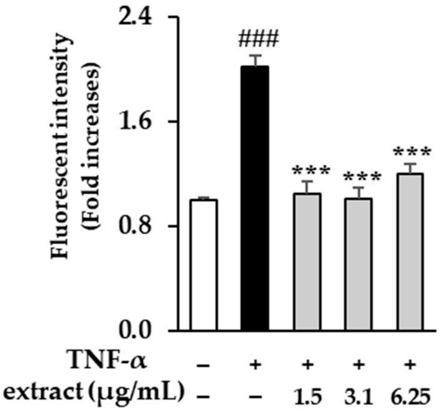

Figure S30. Effects of the extract on TNF-α-induced intercellular reactive oxygen species (ROS) generation. Data are presented as the mean ± SEM (n = 3). ### $p < 0.001$ compared to the control group; *** $p < 0.001$ compared to the TNF-α-treated group.

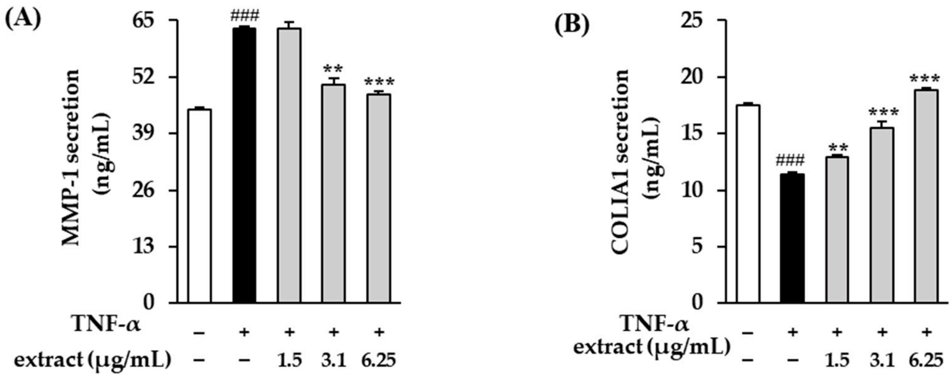

Figure S31. Effect of the extract on MMP-1 and COLIA1 protein expression in tumor necrosis factor-α (TNF-α)-stimulated normal human dermal fibroblasts (NHDFs). Data are presented as the mean ± SEM (n = 3). ### p < 0.001 compared to the control group; ** p < 0.01, and *** p < 0.001, compared to the TNF-α-treated group.
